# An Explainable Transfer Learning Framework Based on Acoustic Spectral Portraits for Pipeline Leak Diagnosis Across Composite Operating and Acquisition Conditions

**DOI:** 10.3390/s26206521

**Published:** 2026-10-14

**Authors:** Zahoor Ahmad, Niamat Ullah, Jong-Myon Kim

**Affiliations:** 1 Department of Electrical, Electronic, and Computer Engineering, University of Ulsan, Ulsan 44610, Republic of Korea; zahooruou@mail.ulsan.ac.kr (Z.A.); niamat016@mail.ulsan.ac.kr (N.U.); 2 Prognosis and Diagnostics Technologies Co., Ltd., Ulsan 44610, Republic of Korea

**Keywords:** acoustic emission, explainable AI, fluid pipelines, leak detection, transfer learning, cross-domain diagnosis

## Abstract

This study proposes an explainable and lightweight pipeline leak-diagnosis framework based on a leak-oriented Acoustic Spectral Portrait (ASP) processing pipeline and transfer learning. The ASP processing pipeline converts one-dimensional acoustic emission signals into compact two-dimensional spectral representations through AR pre-whitening, overlapping Hann-window segmentation, zero-padded fast Fourier transform analysis, logarithmic scaling, percentile-based normalization, and temporal spectral aggregation. These spectral portraits are processed by a lightweight convolutional neural network, in which source-domain knowledge is adapted using limited labeled target-domain data. Gradient-weighted class activation mapping is further employed to provide qualitative post hoc interpretation of the spectral regions contributing to model predictions, rather than direct physical validation, supported by supplementary perturbation, model-randomization, and attribution-stability analyses. The framework was evaluated using three gas- and water-pipeline datasets through six directed source-to-target transfer tasks, with each experiment repeated 10 times using mutually exclusive data partitions. Scenario-averaged classification accuracies of 98.36%, 97.09%, and 98.58% were achieved across the three transfer scenarios. For the representative DS-1 to DS-2 transfer task, transfer learning improved target-domain accuracy from 91.80% to 96.85% while reducing target-model training time from 17.3 min for training from scratch to 9.2 min for fine-tuning. The proposed spectral portrait representation also achieved a mean scenario-averaged classification accuracy of 98.01%, outperforming conventional time-frequency representations. These results demonstrate effective source-to-target adaptation for pipeline leak diagnosis across the three investigated composite operating and acquisition domains.

## 1. Introduction

Pipelines have become the most cost-effective and safe method for transporting gas and oil due to their unique advantages in convenience, reliability, and efficiency [1]. Nonetheless, pipelines are susceptible to leaks due to natural aging, corrosion, climate change, and human interference [2,3]. Pipeline leaks can lead to considerable financial losses, severe environmental contamination, and major consequences for human life. Over the past few decades, extensive research has focused on pipeline leak-related issues, encompassing dynamic risk assessment, numerical analysis, and leakage detection methods, all aimed at mitigating these hazards [4]. Today, leakage detection has become a critical priority for ensuring the safety of pipeline operations, leading to the development of a significant array of methods based on diverse techniques for identifying pipeline leaks. Pipeline leakage behavior has also been investigated through numerical analysis to examine the influence of multiple operating and physical factors on gas leakage and diffusion characteristics [5]. Recent advances in deep learning have also led to the development of a wide range of neural network architectures with different computational and representational characteristics [6].

AE-based monitoring has also been applied to data-driven condition evaluation, demonstrating the usefulness of AE signals for intelligent diagnostic and condition-monitoring systems [7]. AE detects pipeline leaks by recording the transient elastic waves generated when high-pressure fluids escape through small openings in the pipeline. Related research has investigated AE wave propagation and attenuation in concrete using lattice-based modeling, providing additional insights into the propagation characteristics of acoustic signals [8]. In addition, recent studies have demonstrated that deep-learning techniques can effectively characterize transient AE events, further expanding the potential of AE-based monitoring and diagnosis [9]. It is important to acknowledge that pipelines operate under various working conditions, encompassing the surrounding environment, including air, soil, and water, as well as the type of transporting medium (liquid and gas) [10,11]. In the current research work, the primary focus has been on detecting pipeline leaks in specific environments, such as air, water, and soil [1,12,13]. Advanced data-driven approaches have also been investigated for characterizing complex gas–liquid two-phase flow behavior, demonstrating the potential of deep-learning-based feature extraction for challenging multiphase operating conditions [14]. According to the reviewed literature, researchers developed a technique based on AE for detecting leaks in pipelines within open-air environments, demonstrating that time-frequency analysis can effectively eliminate background noise [15,16]. Mahmutoglu et al. [12] effectively identified leaks in underwater gas pipelines by utilizing a realistic, time-varying, multi-path underwater acoustic channel. Their research showcased the enhanced performance of the AE technique for detecting pipeline leaks. However, it is important to note that this approach is limited to a specific working environment. Nonetheless, numerous pipelines extend across multiple working environments over brief distances, with the pipeline in beach oilfields serving as a notable illustration [17]. The research on leakage detection techniques for pipelines remains inadequate due to the diverse challenges presented by various environments [18]. Similarly, the study of leakage detection in gas–liquid two-phase flow, which is a prevalent transmission medium in the oil and gas industry, has received relatively little attention. This is largely attributable to the difficulties associated with feature extraction and recognition. Therefore, it is important to investigate leak-diagnosis techniques capable of maintaining reliable performance across different transported media and operating conditions. Beyond conventional acoustic emission-based approaches, information-theoretic methods have also been investigated for structural damage assessment. Ceravolo et al. explored entropy-based approaches for multiple-damage detection and localization [19] and also investigated information-theoretic entropy for damage detection and localization in buried pipelines [20].

Due to the non-stationary and nonlinear characteristics of AE signals, conventional signal-processing methods may have difficulty extracting sufficiently discriminative leak characteristics under varying operating conditions [21]. Therefore, techniques for pattern recognition have been utilized to identify leaks in pipelines. Over the last twenty years, pattern recognition using machine learning (ML) has experienced significant growth due to advancements in hardware and algorithms, including Artificial Neural Networks (ANNs) and Support Vector Machines (SVMs) [22]. For pipeline leakage detection, Sun et al. [23] integrated SVM with signal processing techniques, including wavelet packet energy analysis, principal component analysis, and local mean decomposition. Leak detection in pipelines using AE methods has also demonstrated the effectiveness of ANN-based classification [24,25,26]. However, a major limitation of the previously mentioned shallow machine learning techniques is that their precision largely depends on features that are extracted manually [27].

Deep Learning (DL) can automatically extract complex features from the signal, which may address the previous challenges faced by shallow ML methods [24]. AE-based pipeline monitoring has also been combined with multi-sensor fusion and data-driven modeling to improve the detection and localization of gas leaks [28]. Song et al. [29] applied CNNs to identify leaks in gas steel pipes, while Heng et al. [30] utilized the same method to pinpoint leaks in water pipes. To achieve an effective deep learning model, two essential conditions need to be met [31]. The first condition involves having a substantial quantity of labeled data for training the DL model, which in turn requires increased training parameters and longer training durations, rendering the training process quite time-intensive [30]. The second requirement is a computer equipped with a powerful central processing unit and a graphics processing unit [32]. Furthermore, implementing DL models presents additional challenges, including adjusting hyperparameters and designing network architecture. In the past few years, transfer learning (TL), a method for transferring models, has been proposed to tackle the aforementioned challenges of DL. In recent years, transfer learning (TL) has been increasingly explored in fault-diagnosis applications to improve the adaptability of deep-learning models across different operating conditions [33]. Recent studies have further demonstrated the application of deep-learning-based methods to AE-event characterization, pipeline leakage diagnosis under varying working conditions, and flow-state prediction in gas pipeline networks [9,34,35]. CNN-based transfer learning has also been successfully applied to complex signal-classification problems beyond industrial monitoring, demonstrating its broader applicability to data-driven diagnostic tasks [36]. Zhou et al. [37] proposed a deep residual network for acoustic emission source localization in steel-concrete composite structures using CWT-based time-frequency representations. Their study demonstrated the potential of deep learning for AE source localization under different structural damage conditions, sensor-to-source distances, and sensor mounting positions. While their work focused on AE source localization in composite structures, the present study investigates pipeline leak diagnosis across composite operating and acquisition conditions using a leak-oriented ASP processing pipeline and CNN-based transfer learning with limited labeled target-domain data. Furthermore, the application of transfer learning to AE-based pipeline leak diagnosis across substantially different operating and acquisition conditions remains comparatively limited.

Despite recent progress in AE-based pipeline leak diagnosis, several challenges remain. First, many existing approaches rely either on handcrafted features or conventional time-frequency representations whose diagnostic characteristics may vary considerably when the operating and acquisition conditions change. Second, deep-learning models trained under one operating condition may exhibit reduced performance when applied directly to a different domain, particularly when only limited labeled target-domain data are available. Third, although transfer learning has been increasingly adopted for fault-diagnosis applications, comparatively few studies have examined AE-based pipeline leak diagnosis across composite source-to-target domain shifts involving simultaneous changes in transported medium, pressure, leak size, pipeline arrangement, and measurement configuration. Finally, the decision processes of deep models are often difficult to inspect, motivating the use of post hoc visualization techniques to identify the spectral regions contributing to individual predictions.

To address these challenges, this study develops an ASP-CNN-TL framework for pipeline leak diagnosis across distinct operating and acquisition domains. The proposed ASP provides a compact leak-oriented two-dimensional representation through AR pre-whitening, overlapping segmentation, zero-padded FFT analysis, logarithmic scaling, and percentile-based normalization. A lightweight CNN is subsequently trained on a selected source domain and adapted using limited labeled data from a target domain. The framework is evaluated through six directed source-to-target transfer tasks, repeated experiments, comparison with conventional signal representations and established deep-learning architectures, and component-ablation analysis. Grad-CAM is additionally employed as a post hoc visualization tool to provide qualitative insight into the ASP regions influencing the CNN predictions.

The novelty and contributions of this work are as follows:

1. A leak-oriented ASP processing pipeline is developed to construct compact two-dimensional representations of pipeline AE signals. The pipeline integrates AR pre-whitening, overlap-preserving Hann-window spectral analysis, zero-padded FFT computation, logarithmic scaling, percentile-based normalization, and temporal spectral aggregation. The methodological contribution lies in the coordinated integration and configuration of these established operations for cross-domain AE-based pipeline leak diagnosis rather than introducing a fundamentally new spectral transform. The contribution of the resulting ASP is evaluated through controlled representation comparisons, component-level ablation, and parameter-sensitivity analyses.
2. A lightweight ASP-CNN-TL framework is developed to transfer source-domain leak-sensitive spectral representations to target operating domains using limited labeled target data. The transfer strategy preserves low-level spectral features while adapting higher-level representations to the target domain.
3. Cross-domain diagnostic performance is systematically evaluated through six directed source-to-target transfer tasks involving three composite operating and acquisition domains. Each experiment is repeated ten times using leakage-safe and mutually exclusive data partitions, and the proposed framework is compared with the same CNN trained without transfer learning and with established deep-learning reference models.
4. The proposed lightweight CNN architecture comprises approximately 2.47 million parameters and requires 274.19 million FLOPs per forward pass. The complete framework achieved a measured processing time of approximately 125 ms per one-second AE recording, including ASP generation and CNN inference, demonstrating computational feasibility under the investigated experimental conditions.
5. The proposed ASP-CNN-TL framework is experimentally evaluated using AE data acquired from pipeline test facilities under distinct gas- and water-pipeline operating conditions.

The remainder of the manuscript is structured as follows: Section 2 provides a detailed explanation of the proposed model and its foundational concepts. Section 3 outlines the experimental setup and presents the results of an ablation study performed to assess the model’s robustness. The final section offers a conclusion summarizing the overall findings of the model.

## 2. Proposed Method

**Figure 1:**
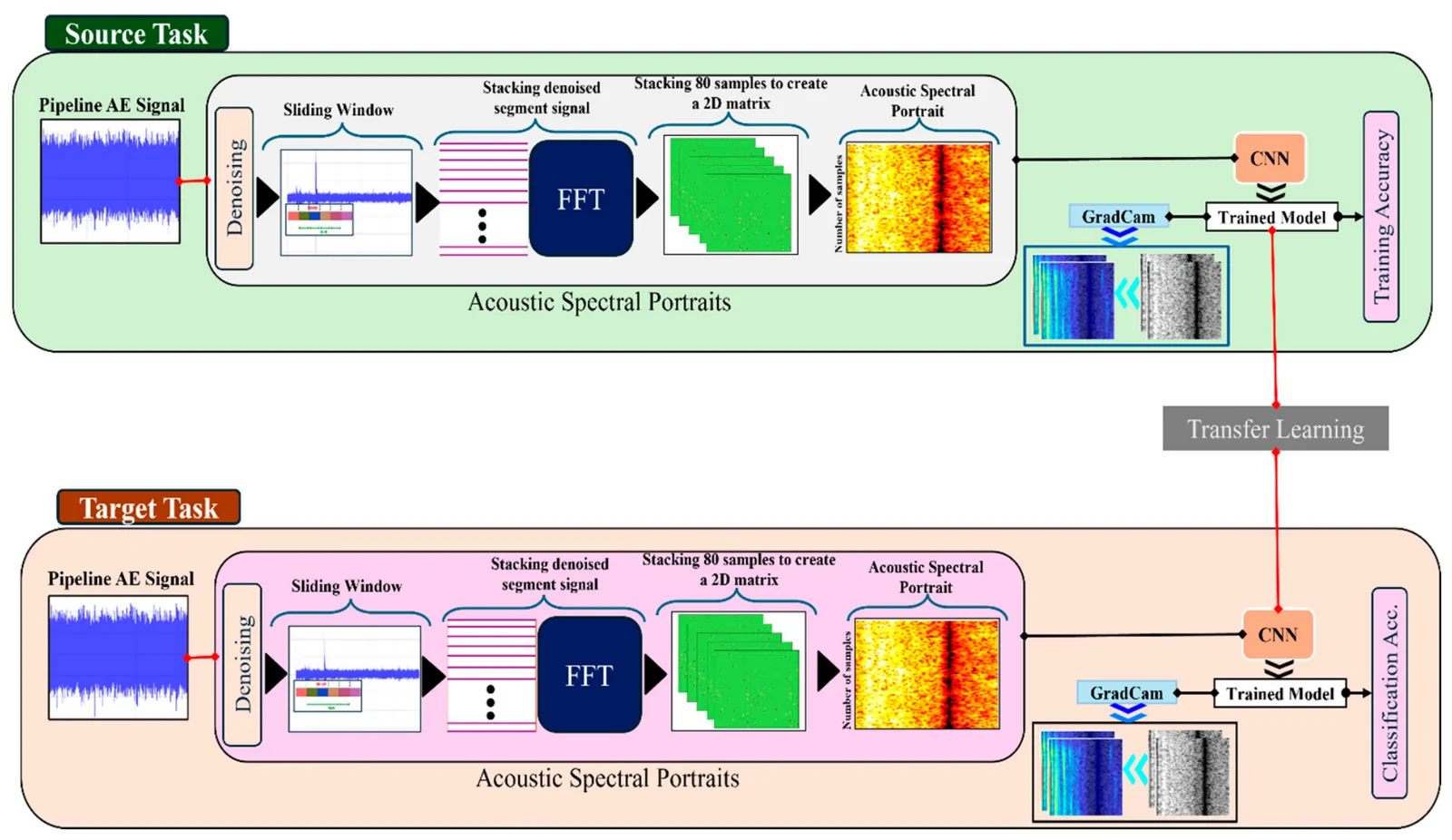
Detailed architecture diagram of the proposed transfer learning-based pipeline leak detection: AE signals are processed into spectral portraits and classified by a CNN, with interpretability provided by Grad-CAM.

The overall architecture of the proposed framework is illustrated in Figure 1. The framework is designed to achieve robust and interpretable pipeline leak diagnosis under varying operating conditions using AE signals. The proposed methodology consists of four major stages: (i) AE signal preprocessing and ASP generation, (ii) deep feature extraction using a CNN, (iii) cross-domain adaptation through transfer learning, and (iv) model interpretability enhancement using Grad-CAM.

Initially, raw AE signals acquired from the pipeline are preprocessed using an AR pre-whitening scheme to suppress environmental noise and stationary spectral disturbances while preserving transient leak-sensitive characteristics. The denoised signals are then segmented through an overlapping sliding-window mechanism combined with Hann windowing to preserve temporal continuity and minimize spectral leakage.

Subsequently, each segmented signal undergoes FFT-based spectral decomposition to obtain localized frequency-domain information. The resulting spectral segments are stacked to construct two-dimensional ASPs, which preserve both temporal progression and frequency-energy evolution of leak-induced acoustic activity. The ASP representation transforms weakly separable one-dimensional AE signals into structured spatial patterns suitable for convolutional feature learning.

The generated ASPs are then used as input to a lightweight convolutional neural network (CNN). For each transfer scenario, the CNN is first trained using a selected source-domain dataset and subsequently adapted to a target-domain dataset through transfer learning. During fine-tuning, the shallow feature-extraction layers are preserved to retain transferable spectral representations, while the deeper layers are updated using limited labeled target-domain data. Because the investigated source and target datasets differ jointly in operating and acquisition characteristics, the transfer process is evaluated under composite domain shifts rather than under isolated variation in a single operating factor.

Finally, Gradient-weighted Class Activation Mapping (Grad-CAM) is incorporated as a post hoc interpretability technique to identify the ASP regions that contribute most strongly to the model predictions. The resulting activation maps provide qualitative insight into the spectral regions used by the CNN when distinguishing normal and leakage conditions. Grad-CAM generates spatial attention heatmaps highlighting the most influential ASP regions contributing to the classification decision, enabling visualization of leak-sensitive spectral characteristics and facilitating physical interpretation of the model predictions.

The proposed framework therefore integrates leak-oriented spectral representation learning, lightweight cross-domain deep feature adaptation, and explainable AI for pipeline leak diagnosis across the investigated composite operating and acquisition domains.

### 2.1. Acoustic Spectral Portrait (ASP) for Pipeline Health Representation

Accurate pipeline leak diagnosis using AE monitoring remains challenging because leak-induced AE signals contain transient and non-stationary components whose spectral characteristics may vary substantially across operating and acquisition conditions. Conventional time-frequency representations, including short-time Fourier transform (STFT) spectrograms and continuous wavelet transform (CWT) scalograms, provide useful descriptions of non-stationary signals; however, direct transformation of the raw AE waveform may retain dominant background components that can obscure comparatively weak transient leakage characteristics. To address this issue, this study develops an Acoustic Spectral Portrait (ASP) representation to emphasize transient spectral variations while generating a compact and consistent two-dimensional representation suitable for CNN-based transfer learning.

**Figure 2:**
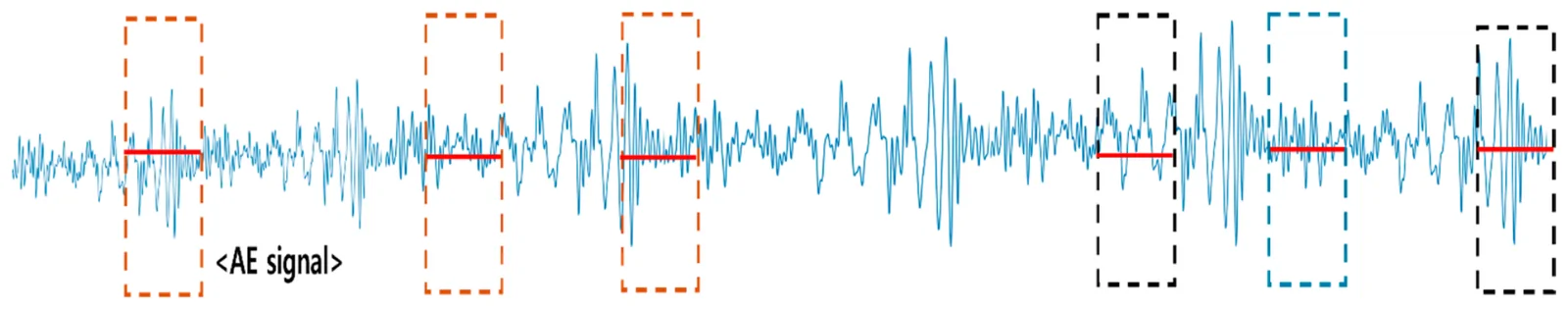
Sliding window technique for the proposed ASP.

**Figure 3:**
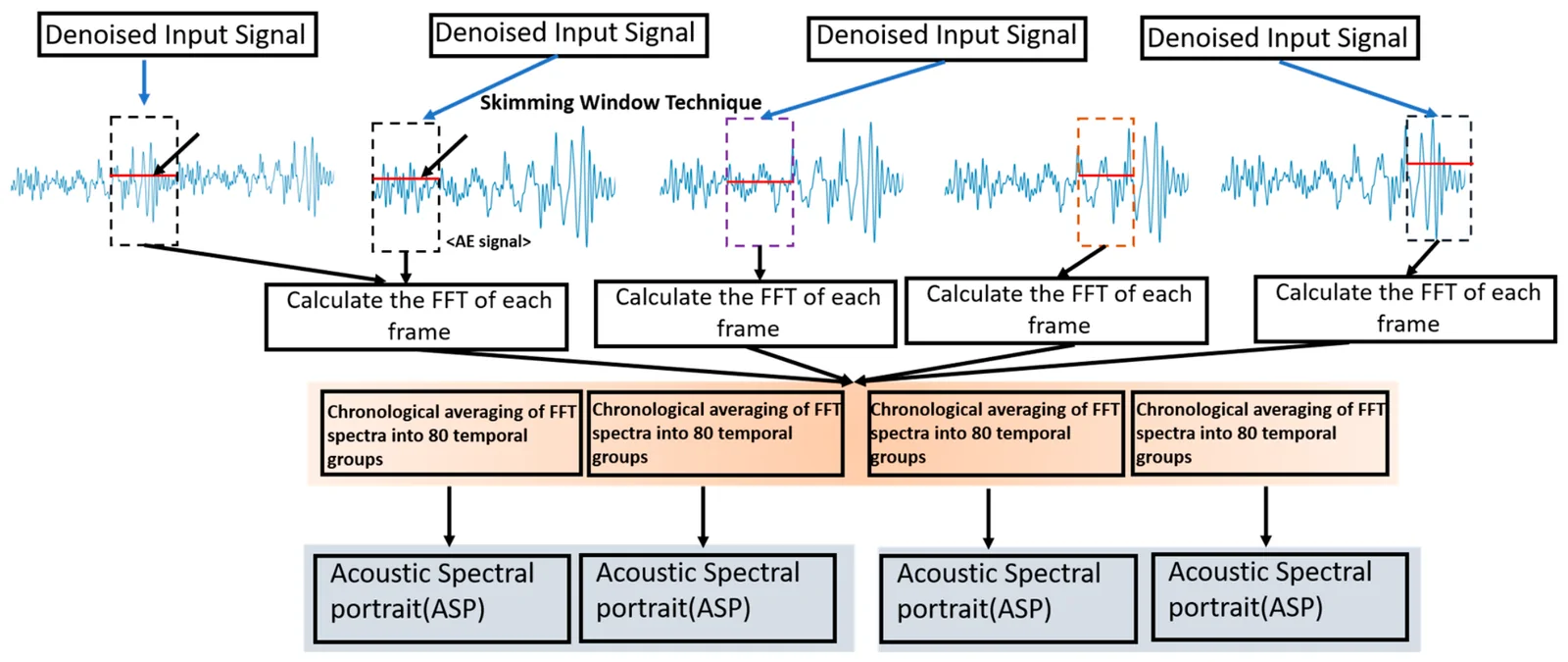
Flow of the Acoustic Spectral Portrait.

The ASP is not introduced as a fundamentally new spectral transform. Rather, its methodological contribution lies in the coordinated integration and configuration of established signal-processing operations to construct a compact, leak-oriented two-dimensional representation for cross-domain AE diagnosis. Specifically, the ASP processing pipeline consists of (i) AR-based pre-whitening to reduce predictable spectral components and retain residual transient activity, (ii) overlapping short-time segmentation with Hann windowing to preserve temporal continuity while controlling spectral leakage, (iii) zero-padded FFT analysis to obtain densely sampled local spectra, (iv) logarithmic scaling and percentile-based normalization to stabilize the spectral representation, and (v) temporal spectral aggregation to organize the densely generated local spectra into a compact representation while preserving their chronological progression. The overall ASP generation procedure, including the overlapping segmentation and final spectral-portrait construction, is illustrated in Figure 2 and Figure 3.

The acquired pipeline AE signal ψ(n) from AE transducers can be expressed as (1).

ψ(n) = *X*(*n*) + *λ*(*n*) + *ω*(*n*) (1)

where X(n) denotes structural vibration components originating from the pipeline, λ(n) denotes leak-induced AE of interest, and ω(n) includes ambient noise and electromagnetic interference. Since leak-induced impulsive components are typically masked by broadband background disturbances, an AR pre-whitening strategy is employed to suppress stationary spectral dominance and amplify transient residual characteristics. The AR process of order K is mathematically expressed in Equation (2).

(2) $$\psi \left(n\right)=\sum_{i=1}^{K}\beta_{i}\psi \left(n-i\right)+\varepsilon \left(n\right),$$

where β_i_ denotes the AR model coefficients, *K* represents the model order, and ε(n) is the prediction residual. Based on the validation-based parameter-sensitivity analysis presented in Ablation Study 4C, the AR model order was set to *K* = 10, which provided the highest validation performance among the evaluated orders. The coefficients are estimated using the Yule–Walker method, after which the predictable component $\hat{\psi }\left(n\right)$ is estimated using Equation (3), and the corresponding pre-whitened residual signal *ψ*_pw_(*n*) is obtained using Equation (4).

(3) $$\hat{\psi }\left(n\right)=\sum_{i=1}^{K}\beta_{i}\psi \left(n-i\right),$$

(4) $$\begin{array}{c} \psi_{\mathrm{pw}}\left(n\right)=\psi \left(n\right)-\hat{\psi }\left(n\right) \end{array}.$$

Unlike conventional amplitude-based denoising, AR pre-whitening estimates the predictable component of each AE recording and retains the corresponding prediction residual for subsequent spectral analysis. This operation reduces the dominance of persistent spectral structures and emphasizes deviations from predictable signal behavior. Because the residual may contain both leak-related transients and other non-stationary components, it is not assumed to represent leakage information exclusively. The contribution of the AR pre-whitening stage is evaluated separately through the component-ablation analysis presented later in this study.

To preserve temporal continuity of transient leak behavior, the denoised signal is partitioned using an overlapping temporal sliding-window mechanism. The total number of segments N_s_ is computed as

(5) $$N_{s}=\left\lfloor \frac{L_{t}-L_{f}}{{∆}_{s}}\right\rfloor +1,$$

where in Equation (5), L_t_ denotes the total signal samples, L_f_ represents the frame length (128 samples), and Δ_s_ indicates the step increment in samples. Based on the sensitivity analysis presented in Ablation Study 4C, an overlap ratio of ρ = 0.5 was selected to provide an effective balance between temporal continuity and redundant spectral information, with the step increment calculated as follows:

∆*_s_* = *Lf* × (1 − *ρ*). (6)

Each segment is subsequently weighted using a Hann window

(7) $$h\left(n\right)=0.5\times \left(1-\cos\left(\frac{2\mathrm{\pi n}}{M-1}\right)\right),\;n=0,1,\ldots ,M-1,$$

where M denotes the window size (M = L_f_ = 128). Compared with rectangular windows, the Hann function provides superior sidelobe attenuation and reduces spectral leakage, thereby preserving weak leak-induced frequency components embedded within high-frequency AE signals. Each pre-whitened segment is multiplied by the Hann window to obtain the corresponding windowed signal, expressed as

*ψ_h_*_(*n*)_ = *ψ*_pw_(*n*) × *h*(*n*). (8)

Here, *ψ*_pw_(*n*) represents the samples of the current pre-whitened frame, and *h*(*n*) is the Hann window defined in Equation (7). Applying the Hann window reduces spectral leakage while preserving the temporal characteristics required for subsequent spectral analysis.

The frequency-domain transformation is then performed using a zero-padded Fast Fourier Transform (FFT) using Equation (9)

(9) $$\Psi \left(k\right)=\sum_{n=0}^{L_{f}-1}\psi_{h}\left(n\right)e^{-j2\mathrm{\pi kn}/N_{\mathrm{FFT}}},$$

where *L_f_* = 128 and *N*_FFT_ = 1024 in Equation (9). Equation (9) sums the 128 Hann-windowed samples, which is mathematically equivalent to appending 896 zeros before performing a 1024-point FFT. Zero-padding does not increase the intrinsic frequency resolution determined by the observation-window duration; rather, it provides denser frequency-bin sampling and a smoother discrete spectral representation. The resulting one-sided spectrum contains 513 frequency bins, including the DC and Nyquist components. The Nyquist-frequency bin was excluded, retaining the first 512 frequency bins for subsequent ASP construction. The power spectral density for each segment is computed using Equation (10).

(10) $$S\left(k\right)=\frac{c_{k}{\left|\Psi \left(k\right)\right|}^{2}}{f_{s}\sum_{n=0}^{L_{f}-1}h^{2}\left(n\right)}$$

Here, *f_s_* = 1 × 10^6^ Hz denotes the sampling frequency, and the denominator incorporates Hann-window energy normalization. The scaling factor is defined as *c*_0_ = 1 and *c_k_* = 2 for 1 ≤ *k* ≤ 511, yielding the one-sided power spectral density (PSD) used for ASP construction. The corresponding FFT-bin spacing is calculated as follows:

(11) $$\Delta f\;=\;\frac{f_{s}}{\mathrm{NFFT}}\;=\;\frac{1,000,000}{1024}\;\approx \;976.56\;\mathrm{Hz}.$$

The PSD vectors obtained from the 15,624 overlapping segments are arranged in their original chronological order to construct an intermediate spectral matrix, expressed as follows:

Θ = [*S*_1_(0) *S*_1_(1) … *S*_1_(511)]      [*S*_2_(0) *S*_2_(1) … *S*_2_(511)]                        [ ⋮ ⋮ ⋱ ⋮ ]                        [*S*_15624_(0) *S*_15624_(1) … *S*_15624_(511)] (12)

Here, *S_m_*(*k*) represents the PSD of the *m*-th overlapping segment at frequency bin *k*, as calculated using Equation (10). Each row of the intermediate spectral matrix represents the PSD of a temporally localized AE segment, with the rows arranged chronologically to preserve the temporal progression and frequency-dependent spectral characteristics of the original AE recording. This intermediate matrix is subsequently subjected to logarithmic scaling, percentile-based normalization, and temporal aggregation to construct the final ASP representation used as input to the CNN.

The PSD values in the intermediate spectral matrix are subsequently transformed into logarithmic space to compress their dynamic range and enhance the representation of comparatively weak spectral components, as expressed in Equation (13).

*Θ_dB_*(*m*,*k*) = 10 × log_10_(*Θ*(*m*,*k*) + *δ*) (13)

where in Equation (13) *m* identifies the temporal segment and *k* identifies the frequency bin, δ = 1 × 10^−10^ prevents numerical instability. Subsequently, percentile-based normalization is applied using the 1st and 99th percentiles as the lower and upper normalization limits, respectively, to suppress extreme spectral outliers while enhancing weak leak-induced patterns. This normalization mechanism improves the consistency of the spectral representation across AE recordings and facilitates more robust transfer learning. The resulting normalized logarithmic spectral vectors are then subjected to chronological temporal aggregation, in which consecutive vectors are grouped and averaged to construct the final 80 × 512 ASP representation.

In comparison with directly using conventional time-frequency representations as CNN inputs, the proposed ASP incorporates AR residual pre-whitening and representation-stabilization operations before constructing a compact spectral matrix. These processing stages are intended to reduce the dominance of persistent spectral components.

**Figure 4:**
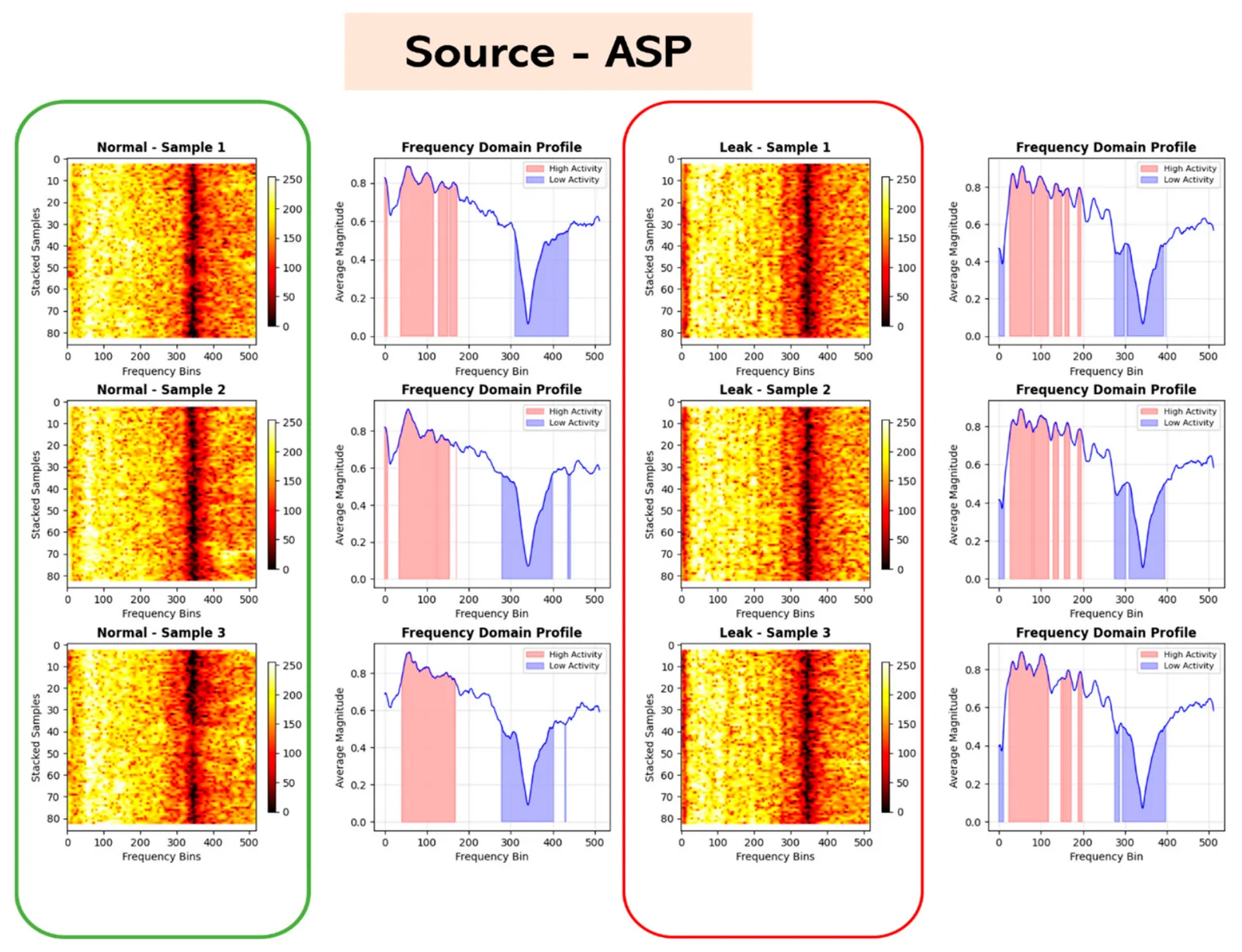
Acoustic Spectral Portraits (ASPs) and corresponding frequency domain profiles of representative source-domain samples. (**Left**): ASP matrices for normal and leak conditions. (**Right**): Averaged frequency magnitude plots highlight high-activity (red) and low-activity (blue) regions used to distinguish between normal and leak states.

**Figure 5:**
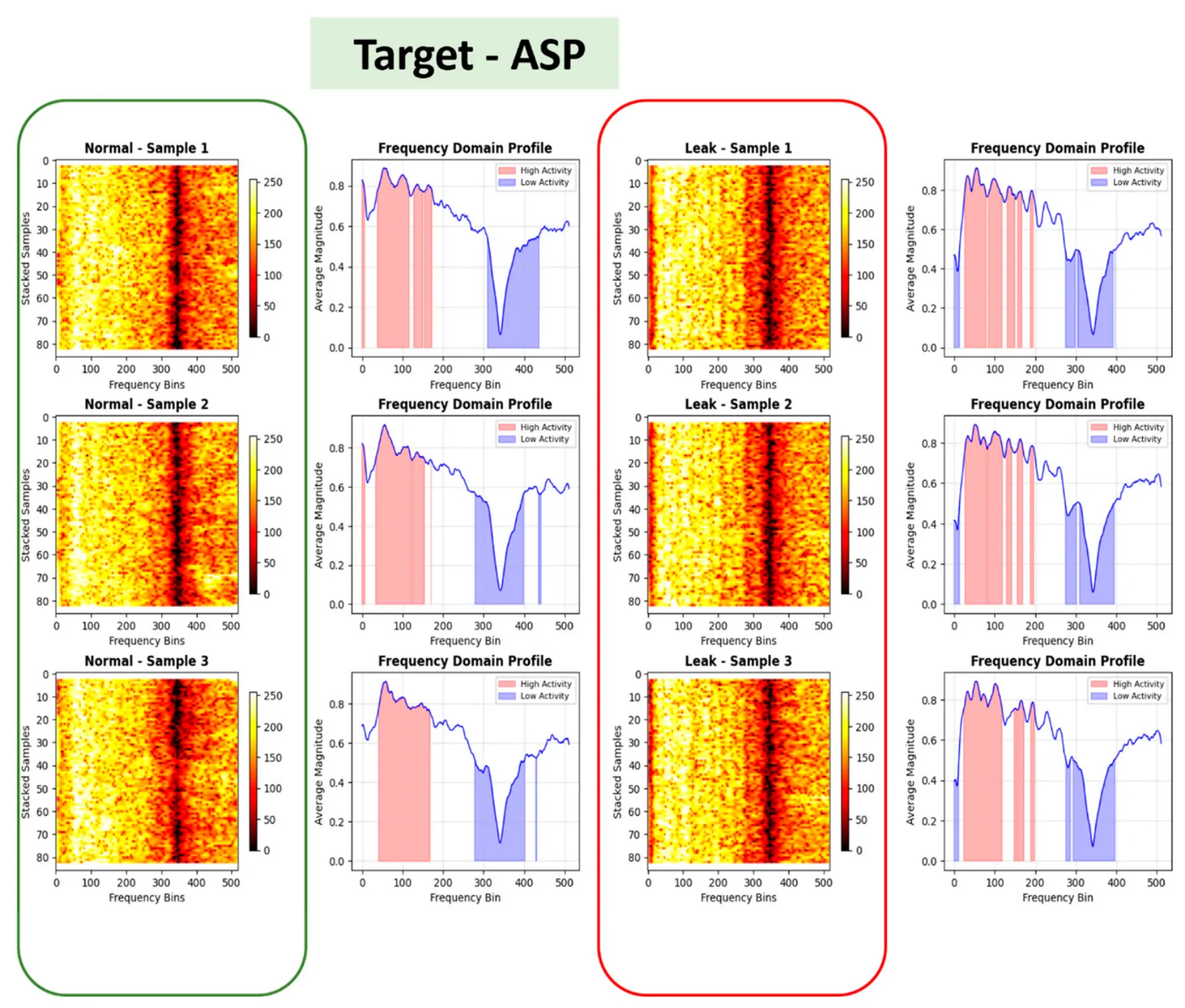
Acoustic Spectral Portraits (ASPs) and corresponding frequency domain profiles of representative target-domain samples. (**Left**): ASP matrices for normal and leak conditions. (**Right**): Averaged frequency magnitude plots highlight high-activity (red) and low-activity (blue) regions used to distinguish between normal and leak states.

While preserving temporally localized spectral activity associated with pipeline leakage. The resulting ASPs exhibit distinct spectral-energy patterns between normal and leaking pipeline conditions, as illustrated in Figure 4 and Figure 5. To determine whether these characteristics translate into improved diagnostic performance, the ASP is further evaluated experimentally against conventional STFT-, CWT-, and synchrosqueezing-based representations using an identical CNN-TL configuration.

Following the spectral-processing stages described above, the final step in ASP construction involves chronological temporal aggregation, which transforms the normalized spectral vectors into a compact two-dimensional representation while preserving information from the complete AE recording. The detailed aggregation procedure is described below.

Each one-second AE recording contains 1,000,000 samples, which produce 15,624 overlapping temporal segments using a 128-sample analysis window with 50% overlap and a step increment of 64 samples. Following PSD computation, logarithmic scaling, and percentile-based normalization, the resulting 15,624 normalized spectral vectors, each containing 512 frequency bins, are maintained in their original chronological order and divided into 80 consecutive, non-overlapping temporal groups of approximately equal size. Specifically, the first 24 groups contain 196 consecutive spectral vectors each, while the remaining 56 groups contain 195 vectors each. Consequently, every spectral vector is assigned to exactly one group, and each group represents approximately 12.5 ms of the original recording.

Within each group, arithmetic averaging is performed independently at each frequency bin across all spectral vectors belonging to that group, following a procedure analogous to temporal coarse-graining. For instance, the values corresponding to the first frequency bin are averaged across all vectors within a given group to produce one representative value. This operation is repeated independently for all 512 frequency bins, generating a single representative spectral vector for each group. The resulting 80 representative vectors are subsequently stacked in their original chronological order to construct the final 80 × 512 ASP representation. Importantly, this procedure incorporates all 15,624 overlapping segments, providing coverage of the complete one-second recording rather than selecting only 80 individual segments. The grouping boundaries are determined exclusively by the chronological positions of the segments, without amplitude-based selection, spectral-energy ranking, or the use of diagnostic class information. The number of temporal groups was determined through the validation-based parameter-sensitivity analysis presented in Ablation Study 4C, where 80 groups provided the highest validation performance among the evaluated configurations. This parameter was subsequently fixed before evaluation on the held-out test data.

### 2.2. Convolutional Neural Network Using Transfer Learning

The proposed diagnostic framework employs a lightweight convolutional neural network integrated with transfer learning (CNN-TL) to evaluate pipeline leak diagnosis across different operating and acquisition domains. Although transfer learning has been widely utilized in conventional image classification and fault-diagnosis applications, its use for AE-based pipeline monitoring under substantial source-to-target distribution shifts remains comparatively limited. In the present study, the source and target datasets may differ jointly in transported medium, operating pressure, leak-orifice size, pipeline arrangement, sensor spacing, and measurement environment. Therefore, the contribution does not lie in transfer learning itself, but in evaluating whether the leak-sensitive spectral representations contained in the proposed ASPs remain sufficiently transferable after adaptation between these composite domains.

**Figure 6:**
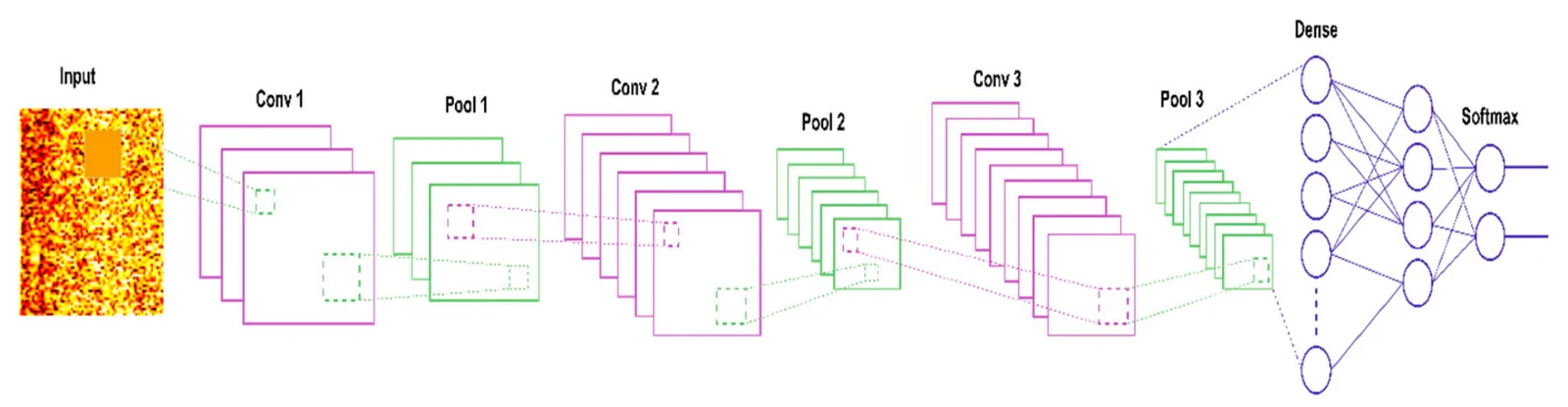
The detailed architecture of the proposed convolutional neural network.

The proposed CNN architecture is specifically designed for compact ASP-based feature learning rather than conventional natural-image analysis. Compared with larger reference architectures, the proposed CNN maintains a relatively compact network structure while preserving strong diagnostic performance. Its measured computational characteristics support the potential for practical pipeline-monitoring applications, although continuous industrial deployment requires further end-to-end validation under representative operating conditions. The detailed architecture of the proposed CNN is shown in Figure 6.

The network consists of three convolutional blocks followed by fully connected classification layers. Each convolutional layer extracts progressively higher-level spatial features from the ASP representation, enabling hierarchical learning of leak-sensitive temporal-frequency patterns. The feedforward operation in a convolutional layer computes feature maps by applying learnable kernels to the output of the preceding layer. To introduce nonlinearity and improve representation capability, the ReLU activation function is employed, as expressed in Equations (14) and (15).

*H^l^* = *f_act_*(*W^l^* ∗ *H^l^*^−1^ + *b^l^*) (14)

*ReLU*(*x*) = max(0,*x*) (15)

The network parameters are optimized using the categorical cross-entropy loss function, which is widely utilized for multi-class classification tasks.

(16) $$L=-\frac{1}{N}\sum_{i=1}^{N}\sum_{c}^{C}y_{\mathrm{true},\mathrm{ic}}\cdot \log\left(y_{\mathrm{out},\mathrm{ic}}\right)$$

Here, *N* is the number of samples, *C* is the number of classes, y_true_ is the true one-hot encoded label, and y_out_ is the predicted probability from the Softmax layer. Parameter updates were performed using the Adam optimizer, a sophisticated gradient-based algorithm chosen for its efficiency and adaptive learning rate capabilities, rather than the traditional stochastic gradient descent (SGD).

For each transfer scenario, one of the three datasets is selected as the source domain, denoted by DS. The CNN is initially optimized using the corresponding source-domain ASPs to learn spectral-spatial representations associated with normal and leakage AE activity. The source-domain optimization objective is formulated as

(17) $$\theta_{S}^{*}={\arg\min}_{\theta_{s}}L_{S}\left(\theta_{S}\right),$$

where θs denotes the optimized source-domain parameters. After source-domain training, the learned weights are transferred to a target-domain model initialized using θs. Depending on the evaluated scenario, DS-1, DS-2, or DS-3 serves as the source domain, while one of the remaining datasets serves as the target domain. The source and target datasets may differ simultaneously in operating and acquisition characteristics; therefore, the transfer task represents adaptation across a composite domain shift. Rather than retraining the entire network from random initialization, the transfer-learning strategy preserves transferable low-level ASP features while adapting higher-level representations to the target dataset.

Specifically, Conv. 1 was frozen during fine-tuning to preserve transferable low-level spectral features, while Conv. 2, Conv. 3, and the fully connected classification layers remained trainable for target-domain adaptation. This strategy reduces computational cost, mitigates overfitting caused by limited target-domain data, and improves adaptation efficiency under varying operating conditions. Fine-tuning is performed using a reduced learning rate of 1 × 10^−4^, a batch size of 32, and 200 epochs to ensure stable convergence while preserving transferable source-domain knowledge.

The target-domain optimization process is expressed as

(18) $$\theta_{T}^{*}={\arg\min}_{\theta_{T}^{\prime }}L_{T}\left(\theta_{S}^{\mathrm{frozen}},\;\theta_{T}^{\prime }\right),$$

where $\theta_{S}^{\mathrm{frozen}}$ represents the frozen feature extraction parameters transferred from the source model, while $\theta_{T}^{\prime }$ denotes the trainable target-specific parameters updated during fine-tuning.

Unlike conventional CNN-based leak-diagnosis approaches that require training from random initialization for a new target condition, the proposed ASP-CNN-TL framework reuses source-trained spectral representations and adapts the higher-level model parameters using limited labeled target-domain data. The experiments therefore evaluate whether ASP-based leak-sensitive characteristics remain sufficiently transferable across the investigated composite operating and acquisition domains. The resulting performance should be interpreted as evidence of successful source-to-target adaptation within the tested datasets rather than as proof of complete domain invariance. The parameters of the ASP-CNN-TL framework are presented in Table 1. The ‘Trainable’ column in Table 1 refers to source-domain training. During source-domain training, all convolutional and fully connected layers are trainable. During target-domain fine-tuning, Conv. 1 is frozen, whereas Conv. 2, Conv. 3, and the fully connected classification layers remain trainable. Pooling and dropout layers contain no trainable parameters.

## 3. Results and Discussion

This section provides a detailed explanation of the experimental testbed for dataset acquisition and certain ablation studies we conducted for the assessment of our proposed ASP-CNN-TL model.

### 3.1. Experimental Testbed

**Figure 7:**
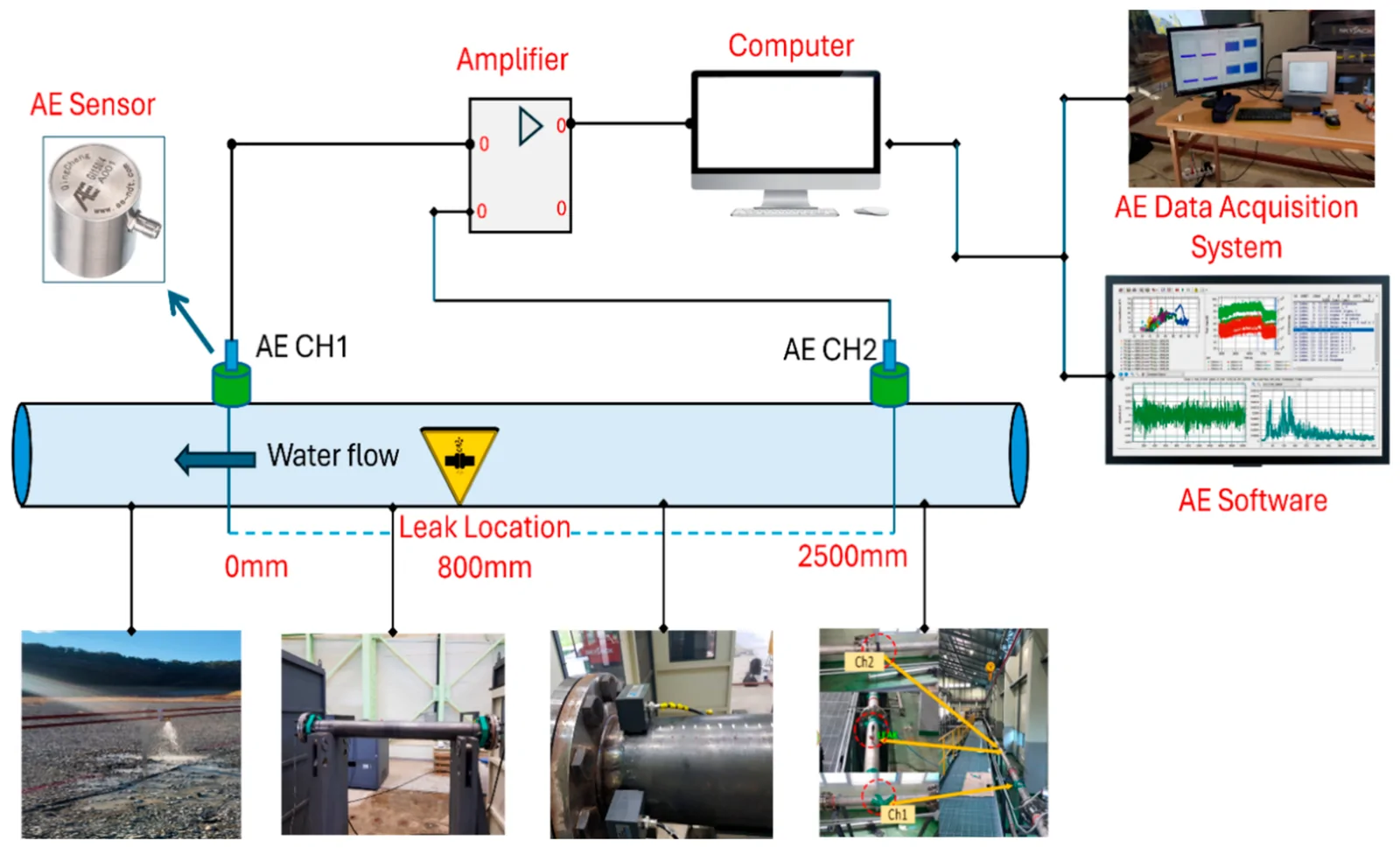
Experimental setup of pipeline leakage AE detection.

To evaluate the proposed method, AE signals were collected from a designated experimental section physically connected to a larger industrial pipeline network. Access to this section was granted for conducting controlled experiments, during which simulated leaks were introduced under specified operating conditions. Figure 7 illustrates the instrumented experimental section rather than the entire industrial pipeline network.

The measurement system employed two MISTRAS R15I-AST acoustic emission sensors equipped with integrated 40 dB preamplifiers and internal filtering circuitry, with a specified operating frequency range of 50–400 kHz. Both sensors were mounted on the pipeline surface, as detailed in Table 2. The same sensors were employed across all three datasets; however, their relative positions differed between the pipeline arrangements. Sensor 1 was used as the reference position (0 mm), while Sensor 2 was positioned 2500 mm from Sensor 1 for DS-1 and 4500 mm for DS-2 and DS-3. The simulated leak position was maintained at 800 mm from Sensor 1. Sensor 2 served as the primary acquisition sensor, whereas Sensor 1, positioned closer to the simulated leak, functioned as a guard sensor to monitor acoustic activity and verify the operation of the sensing system during acquisition. The AE signals were acquired using a National Instruments NI-9223 data-acquisition module, which provides four simultaneous analog-input channels, 16-bit resolution, and internal input signal-conditioning circuitry. The sampling frequency was set to 1 MHz. Accordingly, the 300 normal and 300 leakage recordings reported for each dataset were obtained exclusively from Sensor 2. To improve acoustic coupling, a specialized lubricant was applied between the sensors and the pipeline surface. The sensors were secured using mounting modules and reinforced with industrial tape. Before data acquisition, a Hsu–Nielsen test was performed to qualitatively verify that both sensors produced satisfactory responses; no numerical acceptance threshold was applied. The same sensors were used across all datasets but were installed separately for the indoor gas and outdoor water-pipeline configurations. For DS-1, the sensors remained mounted throughout normal and leakage acquisition. Similarly, for DS-2 and DS-3, the sensors remained mounted on the outdoor pipeline throughout the corresponding measurements. Although sensor repositioning within each experimental configuration was avoided, quantitative mounting-repeatability measurements were not performed. In this research work, pipeline leaks were simulated at a point 800 mm away from CH1 using a tool directly welded onto the pipe. This enables users to control the fluid release with adjustable sizes, thereby simulating leaks of various dimensions (0.3, 1.0, and 2.0 mm). Three distinct pipeline operating conditions were considered in this study. These conditions differed in transported medium, operating pressure, leak-orifice size, pipeline arrangement, and measurement environment. Specifically, the investigated conditions consisted of a 13-bar gas-pipeline condition with a 0.3 mm leak, an 18-bar water-pipeline condition with a 1.0 mm leak, and a 7-bar water-pipeline condition with a 2.0 mm leak. These conditions were subsequently defined as DS-1, DS-2, and DS-3, respectively.

For each experimental condition, the pipeline was initially operated at the specified pressure with the simulated leak closed. Once the operating conditions had stabilized, AE recordings were acquired to characterize the normal state. Subsequently, the corresponding leak was activated, and the operating conditions were allowed to stabilize again before acquiring the leakage-state recordings. Both water-pipeline experiments (DS-2 and DS-3) were conducted at an approximate pipeline flow rate of 54 m^3^/h, with operating pressures of 18 and 7 bar, respectively. The gas-pipeline experiment (DS-1) was conducted at an operating pressure of 13 bar. The leak remained activated throughout the approximately five-minute leakage-state acquisition interval and was subsequently deactivated before establishing another operating condition. The exact stabilization times, individual leakage flow rates, leak-orifice tolerances, and ambient conditions were not systematically recorded.

During each state, 300 separate 1 s AE recordings were acquired at a sampling frequency of 1 MHz, with each recording containing 1 × 10^6^ data points. The recordings were acquired and stored as separate measurement observations rather than being generated by subdividing a longer continuous waveform. Although the nominal operating state remained unchanged during each acquisition interval, successive 1 s AE recordings represent distinct realizations of the measured AE process. Pipeline AE signals are inherently transient and non-stationary because their instantaneous characteristics are influenced by stochastic fluid–structure interactions, turbulent-flow fluctuations, intermittent AE events, multiple propagation paths, reflections, attenuation, interactions with structural discontinuities, and background disturbances. Consequently, the temporal and spectral characteristics of the recorded AE activity can vary substantially between successive observations. However, the 300 recordings obtained for a given state originated from the same stabilized acquisition interval and should therefore not be interpreted as 300 independent physical leak activations or experimental sessions.

Three datasets were generated for assessment according to the leak sizes. The description of the datasets is provided in Table 3. The datasets were acquired from different pipeline operating arrangements. DS-1 corresponds to an indoor gas-pipeline experiment, whereas DS-2 and DS-3 were obtained from an open-space water-pipeline experiment. The same AE sensor type was used for all measurements; however, the sensor mounting positions differed between the gas and water arrangements. Consequently, the datasets represent distinct operating and acquisition conditions rather than numerical modifications of a common measurement dataset. Furthermore, the source and target datasets differ not only in transported medium, operating pressure, and leak size, but also in sensor spacing and acquisition configuration. Accordingly, DS-1, DS-2, and DS-3 are treated in the subsequent transfer-learning experiments as three composite operating and acquisition domains, and the observed domain shift is not attributed to any individual factor in isolation.

**Figure 8:**
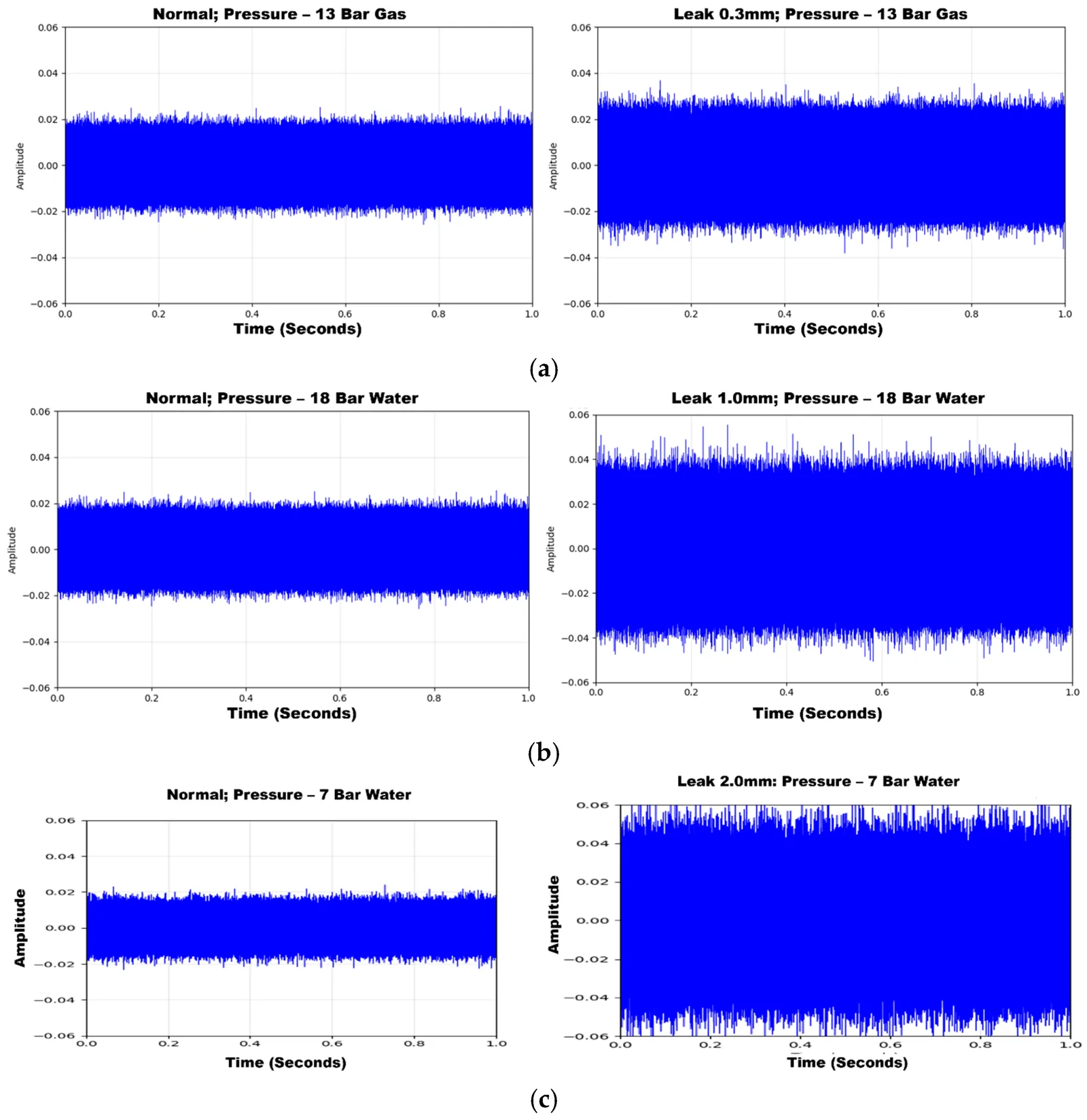
AE signals acquired from the pipeline under various operating conditions: (**a**) 13 bar gas; (**b**) 18 bar fluid pressure; and (**c**) 7 bar fluid pressure.

An AE is a transient sound wave created by a quick release of energy as an elastic wave. This occurs due to changes in the structural integrity of a material, such as that found within a pipeline. The occurrence of a physical phenomenon due to fluid interacting with a structural discontinuity is known as an AE event. AE sensors capture this AE event as a series of AE hits. These hits in the signal may be associated with leaks resulting from fatigue failure, stress fractures, corrosion cracks, and structural irregularities. Therefore, the non-destructive technique that relies on AE is regarded as having a ‘global’ characteristic. The term “global” signifies that AE-based monitoring enables the investigator to obtain a broader understanding of the specimen’s overall performance, regardless of the reasons for its deterioration. In this study, a non-destructive method utilizing AE is employed to examine the condition of the pipeline. Figure 8a–c show the AE signals acquired from the pipeline under different operating conditions, including 13 bar gas pressure, 18 bar water pressure, and 7 bar water pressure, respectively.

Each separately acquired 1 s AE recording constituted one original diagnostic observation. The ASP representation described in Section 2.1 was generated separately for each recording, ensuring that signal information from one observation was not combined with information from another. The resulting ASPs were subsequently assigned to mutually exclusive training, validation, and testing subsets. No original AE recording, or ASP derived from that recording, was shared among these subsets.

#### Experimental Setup

The experiments were conducted on a system featuring a 7th Generation Intel Core™ i7-7700K processor (3.60 GHz, 4 cores/8 threads), 16 GB of RAM, and an NVIDIA^®^ GeForce GTX 1080 Ti GPU with 11 GB GDDR5X memory and 3584 CUDA cores. All algorithms were developed and executed using Python 3.10.11, with Keras and TensorFlow 2.12.0 serving as the primary deep learning frameworks, along with Pandas 1.5.3 for efficient data handling and analysis.

### 3.2. Evaluation of the Diagnostic Capabilities of the Proposed Approach

To evaluate the diagnostic performance of the proposed ASP-CNN-TL framework, six directed source-to-target transfer experiments were conducted across DS-1, DS-2, and DS-3. The ASP representations were used as inputs to the CNN-TL model for binary classification of normal and leakage conditions across the investigated composite operating and acquisition domains.

The model was tested for its diagnostic accuracy using a TL approach, where knowledge from one dataset was applied to others. To reduce the dependence of the reported performance on a single experimental run, each source-to-target experiment was repeated 10 times. For each source-to-target transfer task, the target-domain data were first partitioned in a stratified manner into a 20% development subset and an 80% held-out test subset. The 20% development subset was further divided into 80% training and 20% validation data. Therefore, relative to the complete target-domain dataset, 16% of the samples were used for training, 4% for validation, and 80% for final testing. Because each target dataset contained 600 separately acquired 1 s recordings (300 normal and 300 leakage), this corresponded to 96 training observations, 24 validation observations, and 480 test observations per target-domain experiment while preserving the class distribution. The training and validation subsets formed the target-domain development data and were used exclusively for model fitting and parameter selection. In particular, the candidate values of the ASP parameters *k*, *n*, and *ρ* were evaluated using the development data, with the validation subset used to select the final parameter configuration. The 80% held-out test subset was not accessed during parameter selection and was evaluated only after all ASP parameters and model settings had been fixed. A new stratified partition was generated for each of the 10 repetitions. For reproducibility, the random seeds used for the 10 stratified repetitions were 0, 1, 2, 3, 4, 5, 6, 7, 8, and 9, and the partition generated by each seed was used consistently across all compared methods within the corresponding repetition. Within each repetition, the training, validation, and test subsets were mutually exclusive at the recording level. No original 1 s AE recording, or ASP derived from that recording, was shared among these subsets. Because the recordings corresponding to each operating state were obtained during a single stabilized acquisition interval rather than from multiple independently repeated leak activations, the present partitioning evaluates generalization to unseen 1 s observations within the corresponding acquisition domain. It should not be interpreted as session-level or leak-activation-level validation.

The performance values reported in the subsequent analysis represent the average results obtained over ten repetitions. Because these repetitions use different random partitions of the same recordings, their test subsets overlap and cannot be considered statistically independent. Therefore, the reported mean and standard deviation describe performance variability across repeated evaluations rather than uncertainty across independent physical experiments. Consequently, the results characterize recording-level generalization within the investigated acquisition conditions, while validation across independent acquisition sessions and leak activations remains necessary.

The transfer evaluation was organized into three scenarios. In Scenario 1, DS-2 and DS-3 independently served as source domains, with DS-1 as the target domain. In Scenario 2, DS-1 and DS-3 independently served as source domains, with DS-2 as the target domain. In Scenario 3, DS-1 and DS-2 independently served as source domains, with DS-3 as the target domain. Thus, each dataset served once as the target domain and as the source domain in two separate transfer tasks, resulting in six directed source-to-target evaluations. For performance evaluation, sensitivity (SEN) and specificity (SPE) were calculated to quantify the correct identification of leakage and normal conditions, respectively. Average classification accuracy (ACA) was then determined for each directed transfer task, while scenario-averaged classification accuracy (SACA) was used to summarize the performance of the two transfer tasks within each scenario. These metrics are defined in Equations (19)–(21).

(19) $$\begin{array}{c} \begin{array}{c} \mathrm{Sensitivity}\;\left(\mathrm{SEN}\right)=\frac{\mathrm{TP}}{\mathrm{TP}+\mathrm{FN}}\times 100\%,\\ \mathrm{Specificity}\;\left(\mathrm{SPE}\right)=\frac{\mathrm{TN}}{\mathrm{TN}+\mathrm{FP}}\times 100\%. \end{array} \end{array}$$

(20) $$\begin{array}{c} \mathrm{ACA}=\frac{\mathrm{Sensitivity}+\mathrm{Specificity}}{2} \end{array}$$

(21) $$\mathrm{SACA}=\frac{\sum {\mathrm{SD}}_{\mathrm{ACA}}}{\sum {\mathrm{TS}}_{\mathrm{scenario}}}$$

Here, TP and FN denote correctly and incorrectly classified leakage samples, respectively, while TN and FP represent correctly and incorrectly classified normal samples, respectively. Sensitivity measures the correct identification rate of the leakage class, whereas specificity measures the correct identification rate of the normal class. ACA is calculated as the arithmetic mean of sensitivity and specificity for each directed transfer task. SACA is subsequently calculated by averaging the ACA values obtained from the two transfer tasks within each scenario. In Equation (21), $\sum SD_{\mathrm{ACA}}$ represents the sum of these task-specific ACA values, while $\sum TS_{\mathrm{scenario}}$ denotes the number of transfer tasks within the corresponding scenario, which is two in this study. For each repetition, SACA was calculated by averaging the ACA values of the two corresponding transfer tasks. The scenario-level mean and standard deviation were subsequently calculated from the ten resulting SACA values.

**Figure 9:**
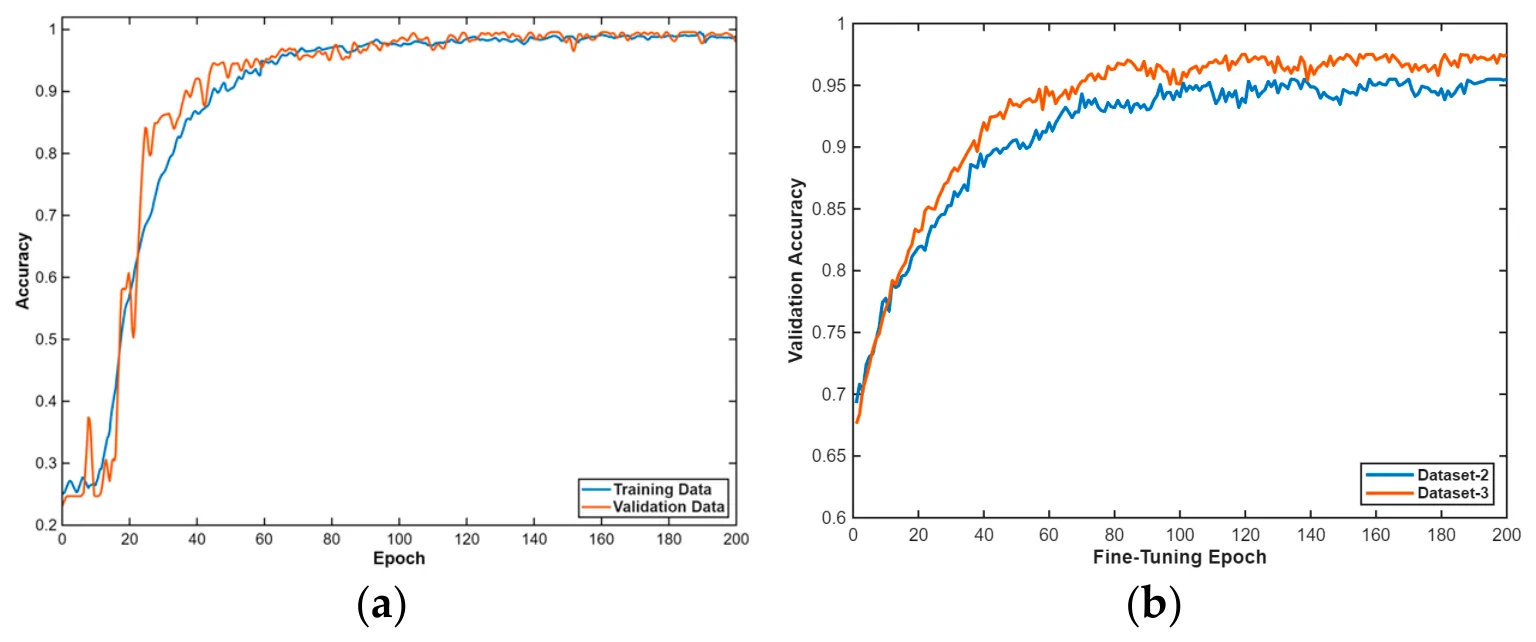
(**a**) Training and validation accuracy curves for source-domain training on DS-1. (**b**) Validation-accuracy curves during target-domain fine-tuning for DS-2 and DS-3.

Based on the average performance obtained over 10 repeated experiments, in Scenario 1, transfer from DS-2 to DS-1 and from DS-3 to DS-1 achieved ACA values of 98.75% and 97.97%, respectively. The specificity was particularly high for the transfer from DS-2 to DS-1, correctly identifying 100% of normal conditions. In Scenario 2, transfer from DS-1 to DS-2 and from DS-3 to DS-2 achieved ACA values of 96.85% and 97.32%, respectively. Scenario 3 yielded the highest overall performance, where transfer from DS-1 to DS-3 and from DS-2 to DS-3 achieved ACA values of 98.90% and 98.26%, respectively. The transfer from DS-1 to DS-3 again achieved 100% specificity for the normal condition. The corresponding SACA values for Scenarios 1, 2, and 3 were 98.36%, 97.09%, and 98.58%, respectively, as summarized in Table 4. These results demonstrate consistent source-to-target adaptation across the investigated composite operating and acquisition domains. The use of a limited 20% target-domain development subset was intended to evaluate the ability of transfer learning to adapt the source-trained model using comparatively limited labeled target data. To improve classification accuracy, 200 fine-tuning epochs were used. Figure 9a shows the training and validation accuracy curves for source-domain training on DS-1, whereas Figure 9b shows the validation-accuracy curves during target-domain fine-tuning for DS-2 and DS-3. Model checkpoints were selected based on the minimum validation loss, and the held-out test sets were evaluated only after the final model configuration and checkpoint had been fixed.

**Figure 10:**
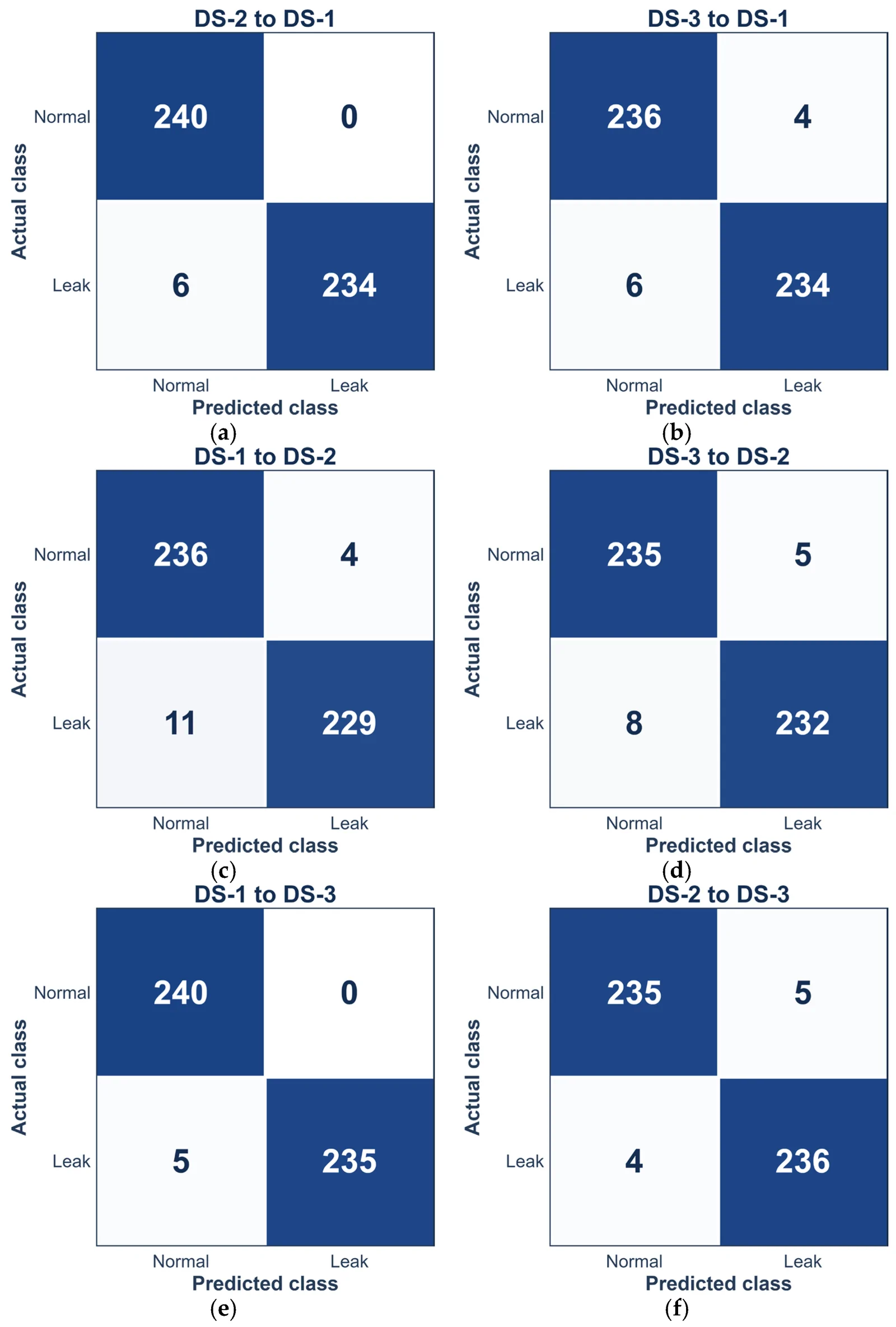
Representative confusion matrices of the proposed ASP-CNN-TL framework for six directed source-to-target transfer tasks: (**a**) DS-2 to DS-1, (**b**) DS-3 to DS-1, (**c**) DS-1 to DS-2, (**d**) DS-3 to DS-2, (**e**) DS-1 to DS-3, and (**f**) DS-2 to DS-3. Each matrix illustrates the classification outcomes of an individual test repetition comprising 240 normal and 240 leakage recordings.

The results indicate that the learned leak-sensitive representations remain useful after adaptation between the investigated datasets despite substantial changes in operating and acquisition conditions. However, because the transported medium, pressure, leak size, pipeline arrangement, and measurement environment were not varied independently, the effect of each individual factor cannot be isolated. Therefore, the reported performance represents transfer across composite domains rather than factor-specific robustness. To further examine the class-specific diagnostic performance of the proposed framework, Figure 10 presents representative confusion matrices for all six directed transfer tasks. Each matrix illustrates the classification outcomes of an individual test repetition comprising 480 held-out recordings. The diagonal elements represent correctly classified observations, whereas the off-diagonal elements indicate false alarms and missed leakage cases. These representative results complement the mean and standard deviation reported in Table 4, providing additional insight into the classification behavior of the proposed framework across the investigated transfer scenarios.

**Ablation Study 1:** To evaluate the contribution of transfer learning more comprehensively, three target-domain evaluation conditions were compared across all six directed source-to-target transfer tasks: source-only inference, target-domain training from scratch, and TL. In the source-only condition, the source-trained CNN was applied directly to the corresponding target-domain held-out test subset without any target-domain fine-tuning. In the scratch condition, the same CNN architecture was initialized randomly and trained using the same target-domain development data and training budget employed for TL. In the TL condition, the CNN was initialized using the corresponding source-domain weights and fine-tuned using the same target-domain development samples. For each of the 10 repeated experiments, identical target-domain partitions were used across the three conditions to ensure that the comparison reflected the effect of transferred source-domain knowledge rather than differences in data availability or test composition.

As summarized in Table 5, the source-only models achieved ACA values ranging from 78.50% to 90.00% across the six directed transfer tasks, indicating that direct application of a source-trained model to a different target domain resulted in a substantial performance reduction under the investigated composite domain shifts. Training the same CNN from random initialization using the available target-domain development data improved the ACA to 91.80 to 93.55%. In comparison, TL consistently achieved the highest target-domain performance, with ACA values ranging from 96.85% to 98.90%. Relative to scratch training, TL improved ACA by 6.10, 6.07, 5.05, 4.22, 6.70, and 4.71 percentage points for DS-2–DS-1, DS-3–DS-1, DS-1–DS-2, DS-3–DS-2, DS-1–DS-3, and DS-2–DS-3, respectively. These results demonstrate that the performance improvement cannot be attributed solely to the availability of labeled target-domain samples, because the scratch and TL models used the same target-domain development and test data, with the principal difference being initialization from source-domain knowledge.

**Figure 11:**
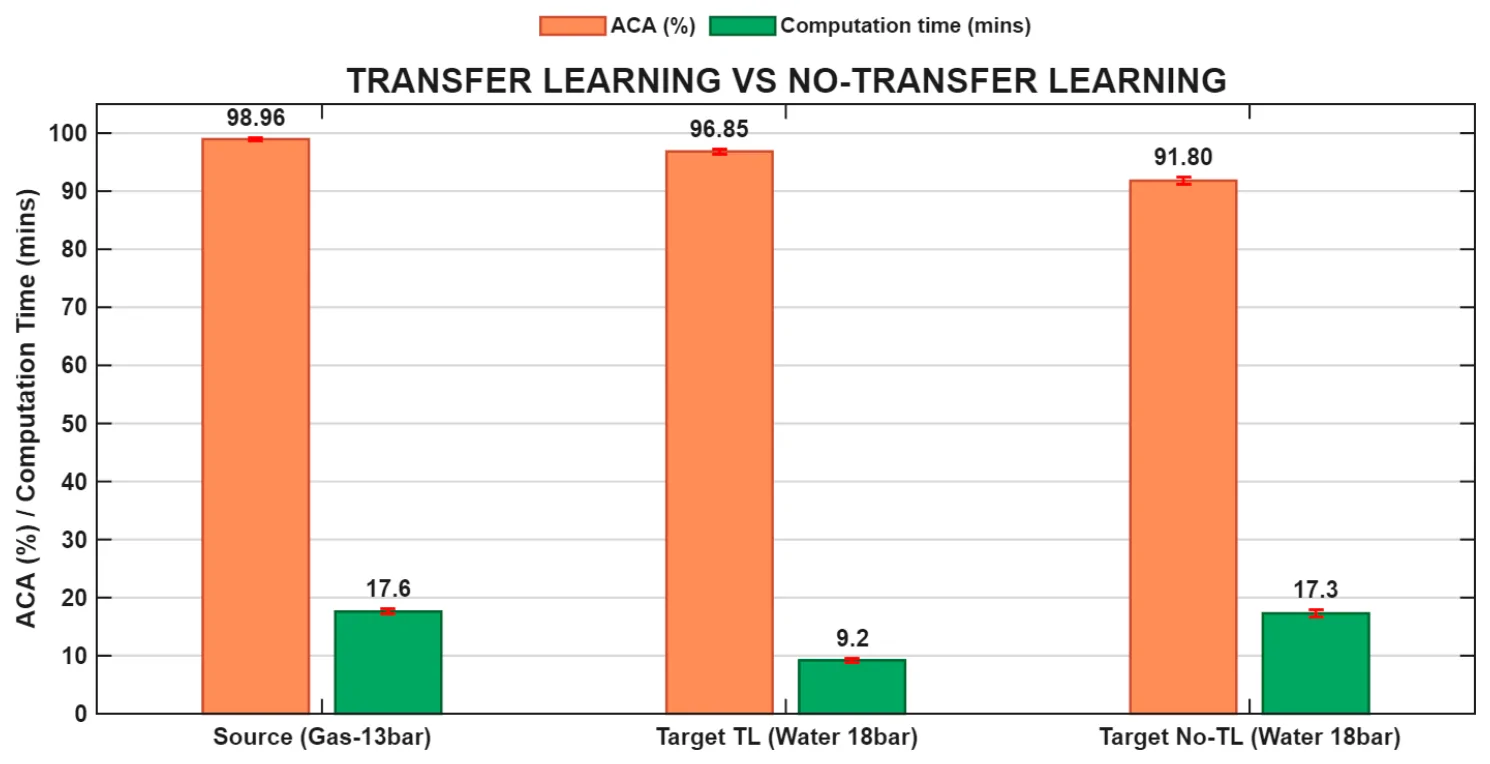
Comparison of the source training model vs. the target TL and target no-TL.

**Figure 12:**
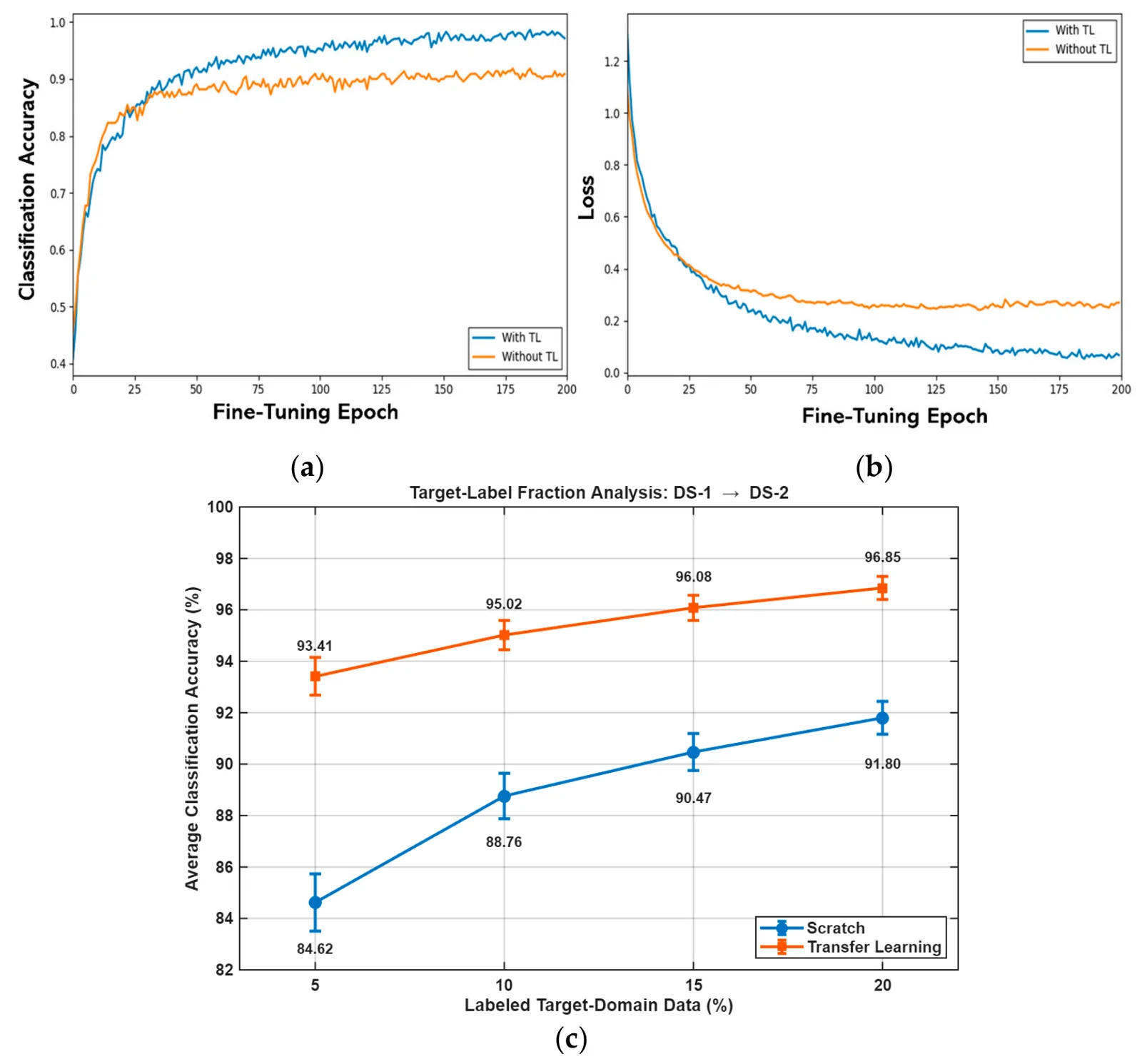
Analysis of the representative DS-1–DS-2 transfer task: (**a**) training/validation accuracy curves for the target models with and without transfer learning; (**b**) corresponding loss curves; and (**c**) target-domain ACA obtained by Scratch and TL under different fractions of labeled target-domain development data. Error bars in (**c**) represent the standard deviation over 10 repeated experiments.

Figure 11 and Figure 12 provide a representative visualization of the DS-1–DS-2 transfer task. For this case, the scratch model achieved an ACA of 91.80 ± 0.63%, whereas the TL model achieved 96.85 ± 0.44%, corresponding to an improvement of 5.05 percentage points. Figure 11 illustrates the corresponding performance comparison for this representative transfer, while Figure 12 shows that the TL model exhibits faster and more stable convergence than training from random initialization. Overall, the three-way comparison across all six transfer directions provides direct evidence that source-domain initialization followed by target-domain fine-tuning improves diagnostic performance beyond both direct source-only inference and target-domain training from scratch.

To further examine the influence of labeled target-domain data availability, an additional label-fraction analysis was conducted using the representative DS-1–DS-2 transfer task. The labeled target-domain development fraction was progressively varied from 5% to 20% of the complete target dataset, corresponding to 24/6, 48/12, 72/18, and 96/24 training/validation observations at 5%, 10%, 15%, and 20%, respectively, while the same held-out target test subset was retained for evaluation. At each fraction, the scratch and TL models were trained using identical target-domain samples and training settings. As shown in Figure 12c, increasing the amount of labeled target-domain data improved the performance of both approaches. At 5% labeled target data, the scratch and TL models achieved ACA values of 84.62 ± 1.10% and 93.41 ± 0.74%, respectively, corresponding to an 8.79-percentage-point improvement with TL. At 10%, the corresponding ACA values increased to 88.76 ± 0.89% and 95.02 ± 0.58%, yielding a 6.26-percentage-point improvement. With 15% labeled target data, the scratch and TL models achieved 90.47 ± 0.72% and 96.08 ± 0.49%, respectively, corresponding to a 5.61-percentage-point improvement. Finally, at the 20% development fraction used in the main experiments, the scratch model achieved 91.80 ± 0.63%, whereas TL achieved 96.85 ± 0.44%, providing a 5.05-percentage-point improvement. These results show that TL consistently outperformed training from random initialization across all evaluated target-label fractions, with the largest benefit observed when labeled target-domain data were most limited.

**Ablation Study 2:** To evaluate the proposed lightweight ASP-CNN-TL configuration, five established reference models were considered: AlexNet, ResNet50, ResNet101, EfficientNetB0, and DenseNet201. These architectures represent different CNN designs and computational requirements. The comparison was conducted using the representative DS-1-to-DS-2 transfer task. Because the reference architectures required architecture-specific spatial resizing and three-channel replication, whereas the proposed CNN operated directly on the original 80 × 512 × 1 ASP representation, the comparison in Table 6 reflects the performance of the complete model–input configurations rather than network architecture alone.

To ensure a consistent initialization procedure, all reference architectures were randomly initialized rather than using ImageNet-pretrained weights. Each model was independently trained on the same source-domain ASP dataset, and its learned weights were subsequently transferred for target-domain adaptation. This approach ensured that all models acquired their initial task-specific knowledge from identical source-domain measurements, allowing their performance to be compared under a common transfer-learning protocol without introducing external pretraining information.

All models received ASP representations generated from the same AE recordings using identical processing parameters. The proposed CNN processed the original 80 × 512 × 1 ASPs, whereas the reference models required resizing to 227 × 227 × 3 for AlexNet and 224 × 224 × 3 for the remaining architectures. The three-channel inputs were obtained by replicating the original ASP channel without introducing additional information. Each reference model’s final classification layer was replaced with a two-class output for normal and leakage conditions. Although architecture-specific resizing was necessary, identical target-domain partitions were maintained across all models during ten repeated experiments. The proposed-model result in Table 6 corresponds to the Target TL experiment in Table 5, and sensitivity consistently refers to the leakage class, treated as positive. Because the reference architectures required architecture-specific spatial resizing and three-channel replication, whereas the proposed CNN operated directly on the original 80 × 512 × 1 ASP representation, the comparison in Table 6 reflects the performance of the complete model–input configurations rather than network architecture alone.

To ensure comparable training conditions, all architectures followed a consistent source-to-target transfer-learning protocol. Following independent source-domain training, the checkpoint achieving the lowest source-validation loss was selected for subsequent target-domain adaptation. During fine-tuning, the initial feature-extraction layers were frozen, while the deeper layers and classification heads remained trainable. Architecture-specific hyperparameters were selected under identical validation-data access and search budgets. Specifically, three candidate learning rates (10^−3^, 10^−4^, and 10^−5^) were evaluated with two architecture-appropriate freezing configurations, providing six candidate configurations per model. Adam optimization, a batch size of 32, and a maximum of 200 fine-tuning epochs were employed. The configuration and checkpoint achieving the lowest target-validation loss were selected independently for each architecture. Identical target-domain partitions were maintained across all models within each of the ten experimental repetitions, and the held-out test data were excluded from model selection.

The candidate freezing configurations and selected learning rates were defined according to the structure of each architecture. For the proposed CNN and AlexNet, freezing either Conv. 1 or Conv. 1–2 was evaluated; freezing Conv. 1 with a learning rate of 10^−4^ was selected for both models. For ResNet50 and ResNet101, the candidate configurations involved freezing the convolutional stem and either the first residual stage or the first two residual stages. Freezing the stem and first residual stage was selected, with learning rates of 10^−4^ and 10^−5^, respectively. For EfficientNetB0, freezing the stem and either the first MBConv stage or the first two MBConv stages was evaluated; freezing the stem and first MBConv stage with a learning rate of 10^−4^ was selected. Similarly, DenseNet201 was evaluated by freezing the stem and either the first dense block or the first two dense blocks. Freezing the stem and first dense block with a learning rate of 10^−5^ was selected. In all configurations, the remaining layers, including the classification heads, were trainable during target-domain adaptation.

**Figure 13:**
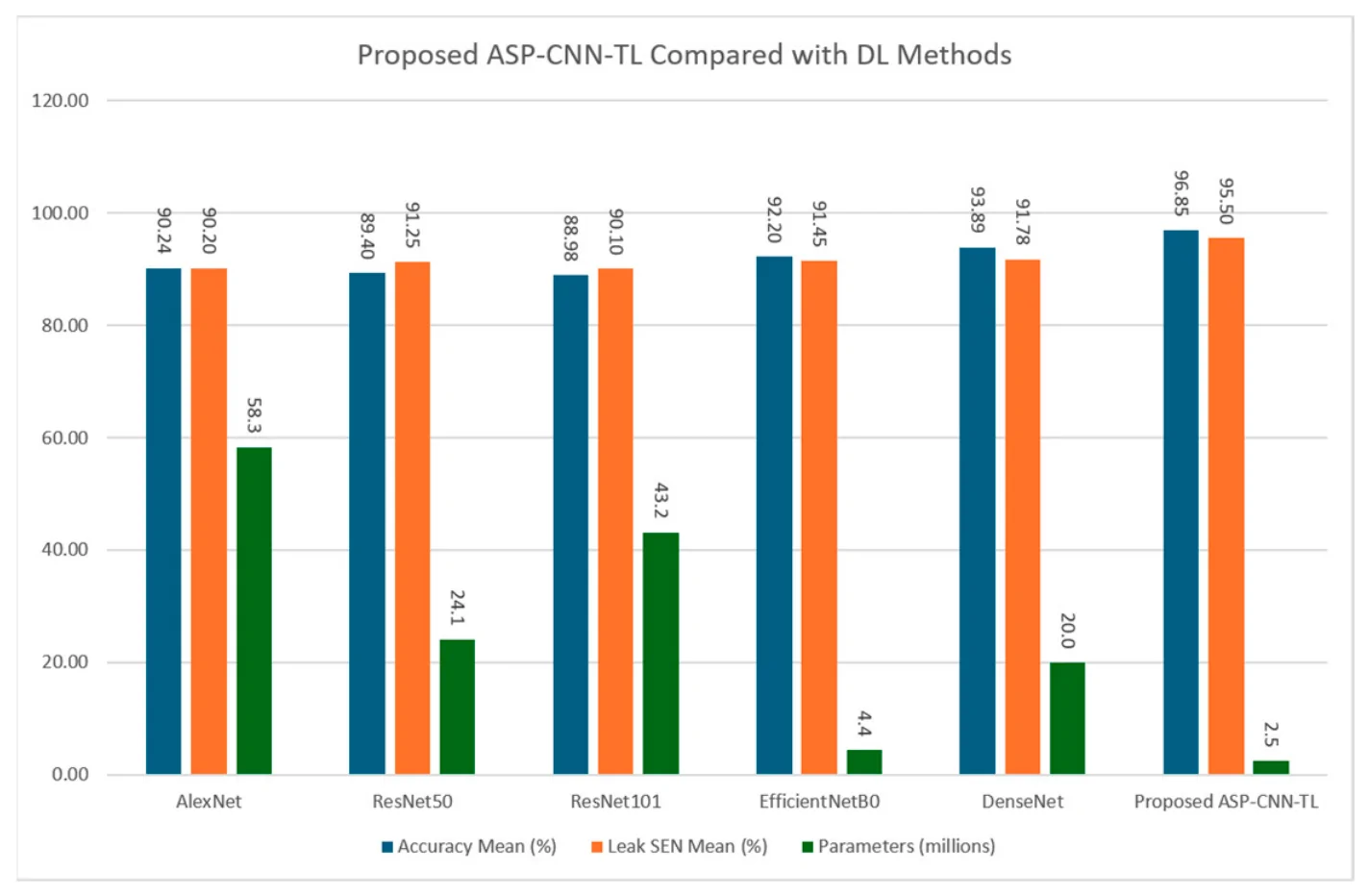
Performance comparison of the proposed ASP-CNN-TL framework with the evaluated deep-learning reference models. The DenseNet architecture shown in the figure refers specifically to DenseNet201.

AlexNet achieved 90.24% accuracy and 90.20% leak sensitivity with 58.3 million parameters. In comparison, the proposed ASP-CNN-TL model achieved 96.85% accuracy and 95.50% leak sensitivity under the same representative DS-1-to-DS-2 transfer setting, corresponding to improvements of 6.61 percentage points in accuracy and 5.30 percentage points in leak sensitivity. The detailed comparison is presented in Table 6 and visually summarized in Figure 13.

ResNet101 achieved 88.98% accuracy and 90.10% leak sensitivity with 43.2 million parameters, whereas the proposed ASP-CNN-TL framework improved these metrics by 7.87 and 5.40 percentage points, respectively, while using approximately 94.3% fewer parameters. EfficientNetB0 achieved 92.20% accuracy and 91.45% leak sensitivity with 4.4 million parameters. The proposed framework exceeded these results by 4.65 percentage points in accuracy and 4.05 percentage points in leak sensitivity, while requiring approximately 43.9% fewer parameters.

Overall, the proposed ASP-CNN-TL framework achieved the highest accuracy and leak sensitivity among the evaluated architectures. Across the evaluated reference models, the improvement ranged from 2.96 to 7.87 percentage points in accuracy and from 3.72 to 5.40 percentage points in leak sensitivity. These results indicate that the proposed configuration provided a favorable balance between diagnostic performance and model complexity under the evaluated transfer-learning setting.

To further characterize the computational requirements of the proposed framework, its architectural complexity, preprocessing time, inference latency, throughput, and memory consumption were evaluated using the experimental hardware described in Section Experimental Setup. The proposed ASP-CNN-TL architecture comprises approximately 2.47 million parameters and requires 137.10 million MACs, corresponding to approximately 274.19 million FLOPs per forward pass, excluding ASP preprocessing. ASP generation required approximately 120 ms per one-second AE recording, while single-record CNN inference required approximately 5 ms, resulting in a combined processing time of approximately 125 ms and a computational throughput of eight recordings per second. For a batch of 32 preprocessed ASPs, CNN inference required approximately 40 ms. The observed peak GPU memory consumption was approximately 0.6 GB for single-record inference and 1.3 GB for batch inference. These measurements distinguish network inference from complete diagnostic processing and demonstrate the contribution of ASP generation to the overall computational workload. The reported inference and memory measurements characterize the proposed framework; corresponding measurements were not obtained for every reference architecture.

**Ablation Study 3:** To further evaluate the diagnostic performance of the proposed ASP-CNN-TL framework, it was compared with study-specific transfer-learning implementations based on the attention-mechanism LSTM (AM-LSTM) architecture of Zhang et al. [38] and the multi-scale one-dimensional CNN (MS1DCNN) architecture of Zhen et al. [39]. The reference architectures were adapted to the present binary leak-diagnosis task by configuring their final classification layers for normal and leakage conditions. For each transfer task, the reference models were first trained on the corresponding source-domain data, after which the source-trained weights were used to initialize the target-domain models. Target-domain adaptation was performed using the same 20% development subset and 80% held-out test split employed for the proposed framework. For consistency across the comparison, Adam optimization with a learning rate of 1 × 10^−4^, a batch size of 32, and 200 fine-tuning epochs was used. All models were evaluated using the same pipeline datasets and the same three transfer-learning scenarios. Each model was evaluated over 10 repeated experiments using identical target-domain partitions within each repetition, and the reported results represent the average performance across the 10 runs. Unlike Table 6, which considers the representative transfer from DS-1 to DS-2, Table 7 provides a scenario-level comparison across the complete transfer-learning scenarios. Accordingly, the proposed-model values in Table 7 correspond to the finalized scenario-level results reported in Table 4.

For the study-specific AM-LSTM implementation, each original one-second AE recording was divided into 80 consecutive, non-overlapping temporal windows. Nine acoustic features were extracted from each window, including RMS, peak amplitude, kurtosis, crest factor, spectral centroid, spectral bandwidth, and three frequency-band powers. The resulting features were chronologically arranged into an 80 × 9 input sequence for each recording. Feature standardization parameters were estimated exclusively from the corresponding training data. The resulting sequences were processed using the LSTM and attention components of the adapted reference architecture, with the classification output configured for normal and leakage conditions.

For the study-specific MS1DCNN implementation, the original one-dimensional AE recordings were used instead of ASP representations or manually extracted features. Each one-second recording containing 1,000,000 samples was divided into 80 consecutive, non-overlapping windows of 12,500 samples. The model processed these windows using its multiscale convolutional architecture and generated a leakage probability for each window. The probabilities were subsequently averaged to obtain a single recording-level prediction, with a threshold of 0.5 used for binary classification. For both reference architectures, data partitioning was performed at the original recording level before window generation or feature extraction. This ensured that information from the same recording was not distributed across the training, validation, and testing subsets. In contrast, the proposed ASP-CNN-TL framework combines a leak-oriented two-dimensional representation of the one-dimensional AE signal with CNN-based transfer learning for diagnosis across composite operating and acquisition conditions.

As summarized in Table 7, the proposed ASP-CNN-TL framework achieved scenario-level classification accuracies of 98.36%, 97.09%, and 98.58% for Scenarios 1, 2, and 3, respectively. These results exceeded those of AM-LSTM-TL by 8.26, 5.24, and 4.87 percentage points, respectively, and those of MS1DCNN-TL by 6.14, 2.75, and 2.73 percentage points, respectively. The proposed framework also required approximately 29–31 min of total training time per scenario, compared with approximately 58–61 min for AM-LSTM-TL and 70–71 min for MS1DCNN-TL. In addition, the proposed model used 2.47 million parameters, compared with 2.2 million for AM-LSTM-TL and 2.8 million for MS1DCNN-TL. Overall, the results in Table 7 indicate that the proposed ASP-CNN-TL framework provided higher scenario-level diagnostic performance while maintaining lower model complexity and shorter training time than the evaluated reference implementations. The reported training times represent different experimental scopes. Specifically, the 9.2 min target-domain fine-tuning time corresponds to the individual DS-1-to-DS-2 transfer task, whereas the scenario-level training times reported in Table 7 represent the combined source-domain training and target-domain fine-tuning durations of the two directed transfer tasks within each scenario. Source-domain training time was measured from the beginning of training until the selected source checkpoint was saved, while target-domain fine-tuning time was measured independently until the corresponding target checkpoint was saved. Both measurements included validation and checkpoint selection but excluded data loading, data conversion, and ASP preprocessing, which was evaluated separately. Therefore, the individual transfer-task and scenario-level measurements characterize different computational workloads.

**Ablation Study 4A—Comparison with conventional spectral representations:** To evaluate the contribution of the proposed ASP representation, STFT, CWT, synchrosqueezed transform (SST), and ASP were compared using the same CNN-TL architecture, source-to-target data partitions, optimization settings, and evaluation protocol. For a more controlled comparison between STFT and ASP, the STFT baseline was constructed using the same 128-sample analysis window, 50% overlap, Hann window, 1024-point FFT, logarithmic spectral scaling, and percentile-based normalization employed in the local spectral-analysis stage of ASP. The conventional STFT was computed directly from the original AE waveform, whereas ASP additionally employed AR pre-whitening before spectral analysis and the proposed temporal spectral aggregation procedure during construction of the final representation. These two operations were intentionally retained as ASP-specific processing stages because incorporating them into the STFT baseline would introduce defining components of the proposed methodology into the reference representation. To ensure compatibility with the same CNN input requirement, the completed STFT representation was subsequently resampled only along its temporal dimension from the original number of short-time frames to 80 uniformly spaced positions using linear interpolation, while the 512-bin frequency dimension was retained unchanged. This dimensional adjustment was performed after construction of the conventional STFT and did not involve the ASP-specific temporal grouping and aggregation procedure.

For the CWT baseline, an analytic Morse wavelet with symmetry parameter 3, time-bandwidth product 60, and 10 voices per octave was used, with scale limits determined automatically from the wavelet support. The SST baseline employed an analytic Morlet wavelet with 32 voices per octave, with scales and frequency limits determined by the associated wavelet filter bank. For both methods, the magnitude coefficient maps were resized by two-dimensional bilinear interpolation to 80 temporal × 512 frequency positions to match the CNN input. This resizing preserved the full time-frequency coverage without applying the ASP-specific temporal grouping and averaging procedure. As shown in Table 8, STFT-CNN-TL achieved scenario-level SACA values of 92.75%, 91.30%, and 93.65% for Scenarios 1–3, respectively. CWT improved these values to 94.15%, 92.90%, and 94.85%, while SST further increased them to 95.25%, 94.15%, and 95.85%. In comparison, the proposed ASP-CNN-TL achieved the highest SACA values of 98.36%, 97.09%, and 98.58%, corresponding to improvements of 5.61, 5.79, and 4.93 percentage points over STFT, 4.21, 4.19, and 3.73 points over CWT, and 3.11, 2.94, and 2.73 points over SST, respectively. The results show a progressive improvement in diagnostic performance from STFT to CWT and SST under the same CNN-TL configuration. In the controlled comparison with STFT, the principal spectral-analysis settings were matched, whereas AR residual pre-whitening and temporal spectral aggregation remained specific to ASP. These ASP-specific stages define the distinct signal-processing and representation-construction strategy evaluated in the proposed framework. The lower SACA standard deviations of ±0.25, ±0.37, and ±0.23 percentage points for Scenarios 1, 2, and 3, respectively, further indicate stable performance across the repeated experiments. Overall, these results suggest that the improved diagnostic performance is associated not only with the CNN-TL classifier but also with the ASP-specific signal-processing and representation-construction strategy.

**Ablation Study 4B—Component-level analysis of the ASP processing pipeline:** To quantify the contribution of the individual ASP processing stages, a one-component-at-a-time ablation analysis was conducted using mean SACA as the evaluation metric. Starting from the complete ASP configuration, each processing stage was individually removed or replaced while all remaining processing settings, CNN-TL architecture, data partitions, optimization parameters, and evaluation conditions were kept unchanged. The complete ASP configuration achieved a mean SACA of 98.01%. Removing the 50% overlap produced the largest performance reduction, decreasing the mean SACA to 95.74%, corresponding to a drop of 2.27 percentage points. Removing AR pre-whitening reduced the mean SACA to 95.92% (2.09 percentage points), indicating that attenuation of predictable background spectral components before local spectral analysis contributes substantially to the final representation. Removing temporal spectral aggregation reduced the mean SACA to 96.27% (1.74 percentage points). In this ablation, the central (98th) spectrum was selected from each of the 80 temporal groups instead of averaging all spectra. This preserved the temporal coverage and 80 × 512 input dimensions while excluding the spectral-averaging operation. This result indicates that the aggregation strategy benefits from information distributed across the complete sequence of densely generated local spectra rather than relying on sparse temporal samples.

Removing logarithmic scaling reduced the mean SACA to 96.52% (1.49 percentage points), while excluding percentile-based normalization resulted in 96.84% (1.17 percentage points). Replacing the Hann window with a rectangular window reduced the mean SACA to 97.18% (0.83 percentage points), indicating a smaller but measurable contribution from spectral-leakage control. Finally, removing zero-padding by using a 128-point FFT instead of the 1024-point FFT resulted in a mean SACA of 97.46% (0.55 percentage points). The comparatively smaller reduction is consistent with the role of zero-padding in providing denser discrete spectral sampling rather than increasing the intrinsic frequency resolution determined by the analysis-window duration. Overall, the results show that the diagnostic performance of ASP arises from the coordinated contribution of multiple processing stages rather than from a single operation.

**Ablation Study 4C—Parameter-sensitivity analysis:** Following the component-level ablation, the sensitivity of ASP to the principal processing parameters was examined using only the target-domain development data, without accessing the held-out test subset. For the AR model order, the mean validation SACA increased from 96.91% at K = 5 to 98.00% at K = 10, before decreasing to 97.42% and 96.78% at K = 15 and K = 20, respectively. These results indicate that K = 10 provided the highest mean validation SACA among the evaluated AR orders. Similarly, increasing the number of temporal aggregation groups from n = 40 (95.86%) to n = 60 (97.14%) and n = 80 (98.00%) progressively improved performance, while further increases to n = 100 (97.63%) and n = 120 (97.18%) provided no additional improvement. Finally, overlap ratios of 0%, 25%, 50%, and 75% produced mean validation SACA values of 95.74%, 97.08%, 98.00%, and 97.36%, respectively. Based on the validation results, K = 10, n = 80, and ρ = 0.5 were adopted for the final ASP configuration and subsequently fixed before evaluation on the held-out test subset.

### 3.3. Grad-CAM Interpretation and Quantitative Attribution Reliability Assessment

Gradient-weighted Class Activation Mapping (Grad-CAM) was employed as a post hoc XAI technique to improve the interpretability of the proposed pipeline leak-diagnosis model. Grad-CAM identifies regions of an input representation that contribute strongly to a selected class prediction [40]. In the present study, it was applied to the ASP inputs to visualize the spectral regions that most strongly influenced the CNN predictions. This is achieved by computing the gradient of the target-class score with respect to the feature activations in the final convolutional layer, producing a spatial map of class-relevant contributions.

Formally, for a target class c, Grad-CAM computes the importance weight $\alpha_{k}^{c}$ for each feature map channel k in the selected convolutional layer, as follows:

(22) $$\alpha_{k}^{c}=\frac{1}{Z}\sum_{i}\sum_{j}\frac{{\partial y}^{c}}{{\partial A}_{\mathrm{ij}}^{k}},$$

where y^c^ is the model output score for class c, ${\partial A}_{\mathrm{ij}}^{k}$ is the activation at spatial location (i,j) of feature map k, and Z is a normalization constant representing the number of spatial positions.

The Grad-CAM map $L_{\mathrm{Grad}-\mathrm{CAM}}^{c}$ is then produced by a weighted linear combination of the feature maps A^k^ using the calculated importance weights, followed by the application of the rectified linear unit (ReLU) to retain only positively correlated features

(23) $$L_{\mathrm{Grad}-\mathrm{CAM}}^{c}=\mathrm{ReLU}\left(\sum_{k}\alpha_{k}^{c}\;A^{k}\right).$$

**Figure 14:**
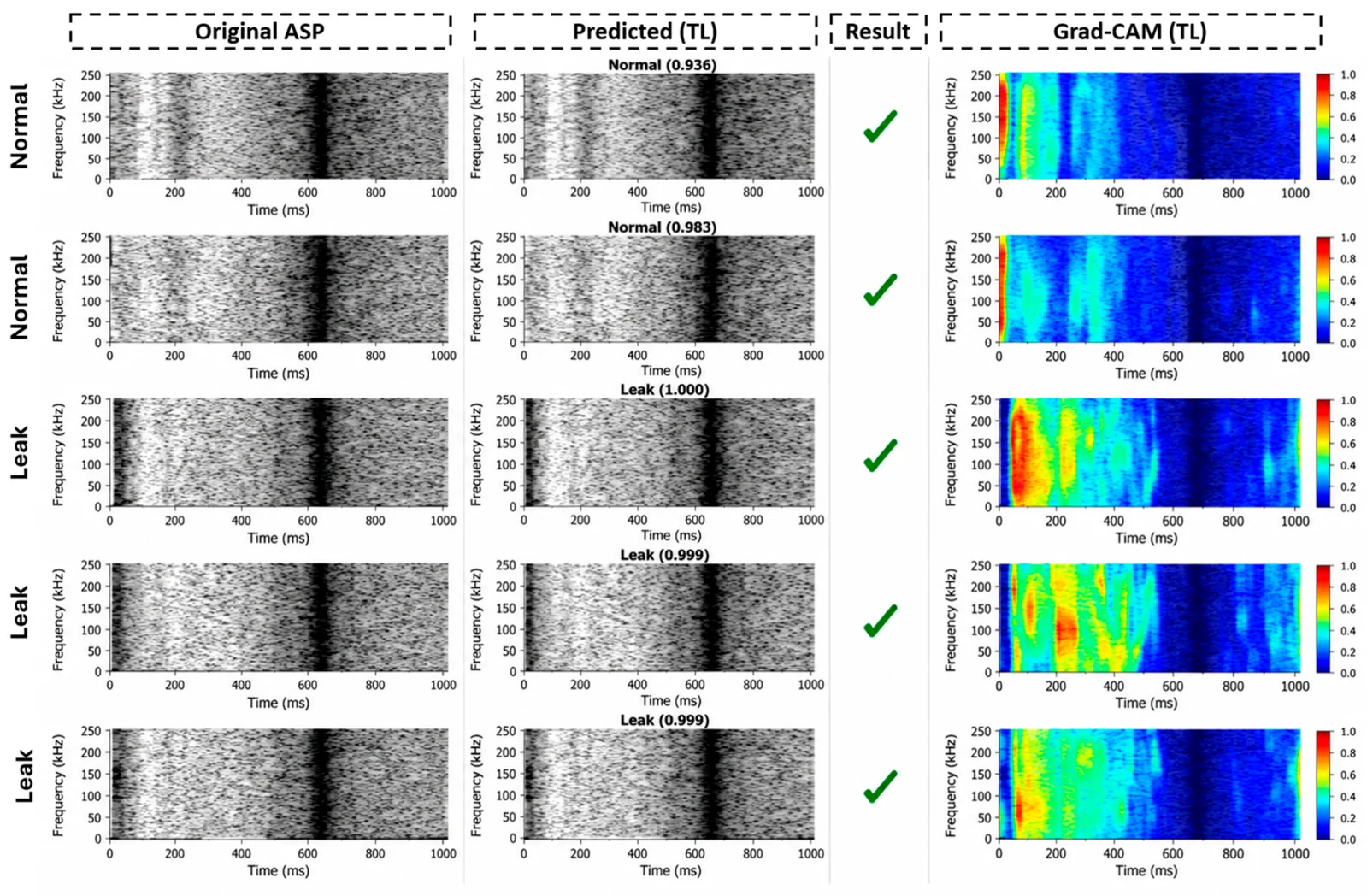
Representative Grad-CAM visualizations for target-domain samples classified using the proposed model with transfer learning. The panels present the original ASP representations, predicted classes and their corresponding probabilities, classification outcomes, and normalized Grad-CAM activation maps. The frequency range of 0–250 kHz is displayed for improved visualization.

**Figure 15:**
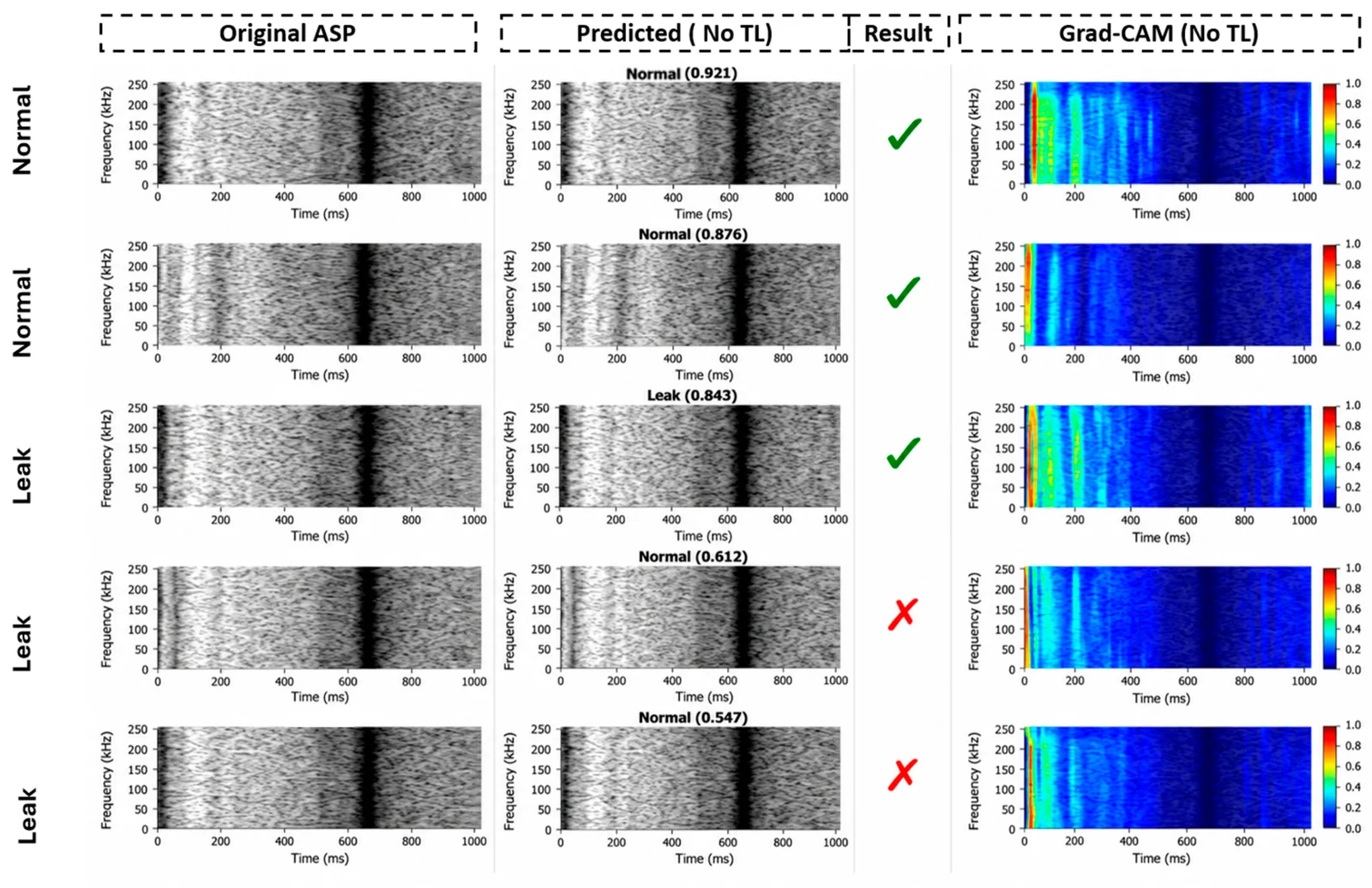
Representative Grad-CAM visualizations for target-domain samples classified using the model without transfer learning. The panels follow the same presentation format as Figure 14, including the original ASP representations, predicted classes and their corresponding probabilities, classification outcomes, and normalized Grad-CAM activation maps. The frequency range of 0–250 kHz is displayed for improved visualization.

Representative Grad-CAM visualizations for the target-domain models with and without transfer learning are presented in Figure 14 and Figure 15, respectively. The revised figures display the original ASP representations, predicted class labels, corresponding predicted-class probabilities, classification outcomes, and Grad-CAM activation maps. The time axis covers the complete 1 s recording, whereas the frequency axis displays a magnified view of the 0–250 kHz range to facilitate visual examination of the attribution patterns. All activation maps are displayed using a normalized color scale ranging from 0 to 1, where warmer colors indicate relatively higher attribution within the corresponding map. Five representative target-domain samples comprising two normal and three leakage examples were selected to illustrate the attribution patterns associated with different classification outcomes. These examples include correctly classified recordings and leakage recordings incorrectly classified by the model trained without transfer learning. The same five recordings were evaluated using both models and presented in the same order to enable direct sample-level comparison. The Grad-CAM visualizations facilitate interpretation of the highlighted regions in terms of their corresponding physical time and frequency coordinates. In the representative examples, relatively strong activations are generally observed during the earlier portions of the recordings, particularly within approximately 0–400 ms. Within the displayed 0–250 kHz frequency range, the distribution of activation varies across individual recordings and classification outcomes, suggesting that different spectral regions contribute to different model decisions.

To quantitatively investigate the reliability of the Grad-CAM explanations, three complementary analyses were conducted: perturbation-based faithfulness evaluation, model-parameter randomization, and attribution-stability assessment.

For the perturbation analysis, the Grad-CAM activation maps were normalized and resized to match the original ASP dimensions. The 10% most and least activated regions were independently identified, and their corresponding ASP values were replaced with frequency-specific median values calculated exclusively from the training data. The original trained CNN was subsequently used to measure changes in the probability of the initially predicted class. For one representative held-out recording (n = 1), masking the 10% most activated regions reduced the predicted-class probability from 0.936 to 0.580, corresponding to a reduction of 0.356. In contrast, masking the 10% least activated regions reduced the probability to 0.900, corresponding to a considerably smaller reduction of 0.036. These findings indicate that the highly activated regions had a greater influence on the selected prediction.

For the model-randomization sanity check, cascading parameter randomization was performed, beginning with the classification layer and subsequently extending to the final convolutional block. The original and randomized Grad-CAM maps were generated using identical recordings and target classes and compared using spatial Spearman rank correlation. For one representative held-out recording (n = 1), the spatial Spearman correlation was 0.09, indicating substantial differences between the original and randomized attribution maps for that recording.

Finally, attribution stability was assessed using the five representative recordings shown in Figure 14 and Figure 15 (two normal and three leakage), evaluated across ten independently trained CNN instances. For each recording, pairwise Spearman correlations were calculated between the corresponding Grad-CAM maps. These correlations were averaged for each recording and subsequently summarized across the evaluated recordings. The analysis yielded a mean spatial Spearman correlation of 0.81 ± 0.07, indicating relatively consistent attribution patterns across the investigated recordings and training repetitions. Collectively, these analyses provide illustrative evidence of attribution relevance, parameter sensitivity, and spatial consistency for the evaluated recordings, rather than establishing dataset-wide attribution reliability.

The Grad-CAM analysis therefore complements the quantitative classification results by providing a visual explanation of which ASP regions contribute most strongly to individual model decisions. However, these activation maps represent model-attribution patterns and should not be interpreted as independent physical validation of the underlying leakage mechanism. Instead, they provide a post hoc qualitative assessment of the spectral information used by the CNN during classification.

## 4. Conclusions

In this study, an explainable and lightweight ASP-CNN-TL framework was developed for AE-based pipeline leak diagnosis across three experimentally investigated pipeline domains that differed jointly in transported medium, operating pressure, leak-orifice size, pipeline arrangement, sensor spacing, and measurement environment. The proposed framework integrated a leak-oriented Acoustic Spectral Portrait, a transfer learning-based convolutional neural network, and Grad-CAM interpretability for computationally efficient and interpretable pipeline leak diagnosis. The Acoustic Spectral Portrait framework transformed one-dimensional AE signals into discriminative two-dimensional spectral representations through AR-based pre-whitening, overlapping temporal segmentation, and FFT-based spectral analysis. Experimental results obtained from the investigated pipeline datasets demonstrated that the proposed framework maintained high diagnostic performance after adaptation across composite operating and acquisition domains involving differences in transported medium, pressure, leak size, pipeline arrangement, and measurement configuration.

The main findings and contributions of this study were summarized as follows:

1. A leak-oriented ASP framework was proposed to preserve transient temporal-frequency characteristics of AE signals and generate discriminative spectral representations suitable for CNN-based feature learning.
2. The proposed ASP-CNN-TL framework achieved effective source-to-target leak diagnosis across the investigated composite operating domains, attaining a scenario-averaged classification accuracy of up to 98.58%.
3. For the representative DS-1→DS-2 transfer task, compared with conventional CNN training without transfer learning, the proposed TL framework improved classification accuracy from 91.80% to 96.85% while reducing training time from 17.3 min to 9.2 min.
4. The proposed lightweight CNN architecture achieved superior performance using only 2.47 million parameters, demonstrating lower computational complexity compared with several established deep-learning models.
5. Grad-CAM provided qualitative post hoc interpretation of the ASP regions influencing CNN predictions. Supplementary perturbation, model-randomization, and attribution-stability analyses provided additional evidence of attribution relevance and consistency under the evaluated conditions. Nevertheless, these attribution patterns should not be interpreted as independent physical validation of the underlying leakage mechanism.

Future work will investigate the performance of the proposed framework under low signal-to-noise ratio conditions and more severe industrial noise environments. In addition, validation will be extended to multiple independently repeated leak activations, acquisition sessions, sensor-remounting conditions, broader leak sizes, and additional pressure conditions. Controlled one-factor-at-a-time experiments will also be considered to evaluate factor-specific robustness. Such experiments will enable session-to-session and installation-to-installation generalization to be evaluated beyond the recording-level generalization investigated in the present study. Future investigations will additionally examine continuous industrial monitoring performance through system-level latency, memory utilization, and computational-efficiency assessments under representative deployment conditions. Future studies will also examine the effects of propagation distance, wave attenuation, and sensor configuration on ASP characteristics.

## Figures and Tables

**Table 1:** Parameters of the proposed ASP-CNN-TL framework.

Layer	Parameters	Output Shape	Trainable	Transferable
Input	Preprocessed Signals	80 × 512 × 1	-	-
Conv. 1	3 × 3 Kernel, 32 Filters, Same Padding	80 × 512 × 32	Yes	Yes
Pool 1	Kernel Size: 3 × 3	27 × 171 × 32	No	Yes
Dropout 1	Rate: 0.25	27 × 171 × 32	No	Yes
Conv. 2	Kernel Size: 3 × 3, Filters: 64, Padding: Same	27 × 171 × 64	Yes	Yes
Pool 2	Kernel Size: 3 × 3	9 × 57 × 64	No	Yes
Conv. 3	Kernel Size: 3 × 3, Filters: 128, Padding: Same	9 × 57 × 128	Yes	Yes
Pool 3	Kernel Size: 2 × 2	5 × 29 × 128	No	Yes
Dense	128 Nodes	128	Yes	Yes
Dropout 2	Rate: 0.5	128	No	Yes
Softmax	2 Nodes (Output)	2	Yes	No

**Table 2:** Parameters utilized for data collection.

No	Parameter	DS-1	DS-2/DS-3
1	Sensor 1 reference position	0 mm	0 mm
2	Sensor 2 position relative to Sensor 1	2500 mm	4500 mm
3	Leak position relative to Sensor 1	800 mm	800 mm
4	Thickness of pipeline	6.02 (mm)	6.02 (mm)
5	Material of the pipeline	304 stainless steel	304 stainless steel
6	Outer diameter of the pipeline	114.3 mm	114.3 mm

**Table 3:** Datasets Description.

Dataset	Arrangement	Medium	Pressure	Health State	Leak Size	Recording Duration	No. of Recordings
Dataset-1 (DS-1)	Indoor Pipeline	Gas	13 bar	Leak/Normal	0.3 mm/Sealed	1 s	300/300
Dataset-2 (DS-2)	Open-space Pipeline	Water	18 bar	Leak/Normal	1.0 mm/Sealed	1 s	300/300
Dataset-3 (DS-3)	Open-space Pipeline	Water	7 bar	Leak/Normal	2.0 mm/Sealed	1 s	300/300

**Table 4:** Diagnostic performance of the proposed model reported as mean ± standard deviation over 10 repeated experiments.

Scenario	Source Dataset	Target Dataset	SEN/SPE (%)		ACA (%)	SACA (%)
			Leak	Normal		
1	DS-2	DS-1	97.50 ± 0.38	100 ± 0.00	98.75 ± 0.19	98.36 ± 0.25
	DS-3		97.70 ± 0.41	98.25 ± 0.35	97.97 ± 0.32	
2	DS-1	DS-2	95.50 ± 0.56	98.20 ± 0.39	96.85 ± 0.44	97.09 ± 0.37
	DS-3		96.74 ± 0.48	97.90 ± 0.36	97.32 ± 0.40	
3	DS-1	DS-3	97.80 ± 0.36	100 ± 0.00	98.90 ± 0.18	98.58 ± 0.23
	DS-2		98.42 ± 0.31	98.10 ± 0.34	98.26 ± 0.29	

**Table 5:** Target-domain performance comparison of source-only inference, training from random initialization (Scratch), and TL across the six directed source-to-target tasks, reported as mean ± standard deviation over 10 repeated experiments.

Source–Target	Source-Only ACA (%)	Scratch ACA (%)	TL ACA (%)	TL Gain vs. Scratch (pp)
DS-2–DS-1	83.40 ± 1.08	92.65 ± 0.71	98.75 ± 0.19	+6.10
DS-3–DS-1	81.90 ± 1.17	91.90 ± 0.76	97.97 ± 0.32	+6.07
DS-1–DS-2	79.20 ± 1.25	91.80 ± 0.63	96.85 ± 0.44	+5.05
DS-3–DS-2	89.10 ± 0.86	93.10 ± 0.65	97.32 ± 0.40	+4.22
DS-1–DS-3	78.50 ± 1.31	92.20 ± 0.69	98.90 ± 0.18	+6.70
DS-2–DS-3	90.00 ± 0.81	93.55 ± 0.61	98.26 ± 0.29	+4.71

**Table 6:** Performance comparison of the proposed ASP-CNN-TL framework and the evaluated deep-learning models reported as mean ± standard deviation over 10 repeated experiments.

Models	Accuracy (%)	Leak SEN (%)	Parameters (Millions)
Alexnet	90.24 ± 0.74	90.2 ± 0.69	58.3
ResNet50	89.4 ± 0.82	91.25 ± 0.71	24.1
ResNet101	88.98 ± 0.88	90.1 ± 0.79	43.2
EfficientNetB0	92.2 ± 0.63	91.45 ± 0.58	4.4
DenseNet201	93.89 ± 0.55	91.78 ± 0.61	20
**Proposed ASP-CNN-TL**	**96.85 ± 0.44**	**95.50 ± 0.56**	**2.47**

**Table 7:** Scenario-level diagnostic performance of the proposed ASP-CNN-TL framework and the reference models, reported as mean ± standard deviation over 10 repeated experiments.

Scenario	Model	SACA (%)	Difference from Proposed (pp)	Parameters (Millions)	Training Time (mins)
1	Attention-LSTM-TL [38]	90.10 ± 0.71	8.26	2.2	60.34 ± 1.18
	MS1DCNN-TL [39]	92.22 ± 0.61	6.14	2.8	70.41 ± 1.34
	**Proposed ASP-CNN-TL**	**98.36 ± 0.25**	**-**	**2.47**	**30.51 ± 0.67**
2	Attention-LSTM-TL [38]	91.85 ± 0.64	5.24	-	58.47 ± 0.12
	MS1DCNN-TL [39]	94.34 ± 0.53	2.75		71.25 ± 1.30
	**Proposed ASP-CNN-TL**	**97.09 ± 0.37**	**-**		**29.46 ± 0.61**
3	Attention-LSTM-TL [38]	93.71 ± 0.57	4.87	-	60.53 ± 1.16
	MS1DCNN-TL [39]	95.85 ± 0.46	2.73		70.36 ± 1.25
	**Proposed ASP-CNN-TL**	**98.58 ± 0.23**	**-**		**31.29 ± 0.64**

**Table 8:** Diagnostic performance of different signal representations using the same CNN-TL framework, reported as mean ± standard deviation over 10 repeated experiments.

Representation	Scenario 1 SACA (%)	Scenario 2 SACA (%)	Scenario 3 SACA (%)	Mean SACA (%)
STFT-CNN-TL	92.75 ± 0.72	91.30 ± 0.79	93.65 ± 0.69	92.57
CWT-CNN-TL	94.15 ± 0.61	92.90 ± 0.67	94.85 ± 0.57	93.97
SST-CNN-TL	95.25 ± 0.51	94.15 ± 0.55	95.85 ± 0.47	95.08
ASP-CNN-TL	98.36 ± 0.25	97.09 ± 0.37	98.58 ± 0.23	98.01

## Data Availability

The data presented in this study are available on request from the corresponding author due to confidentiality and data-sharing restrictions imposed by the industrial partner that provided access to the experimental pipeline facility.
